# Comprehensive analysis of RNA-binding protein SRSF2-dependent alternative splicing signature in malignant proliferation of colorectal carcinoma

**DOI:** 10.1016/j.jbc.2023.102876

**Published:** 2023-01-06

**Authors:** Weizhen Liu, Dongfang Li, Ting Lu, Haosheng Zhang, Zhengxin Chen, Qinli Ruan, Zihui Zheng, Linlin Chen, Jun Guo

**Affiliations:** 1 Key Laboratory of Drug Target and Drug for Degenerative Disease, School of Medicine & Holistic Integrative Medicine, Nanjing University of Chinese Medicine, Nanjing, Jiangsu, China; 2 National Center for Colorectal Diseases, Nanjing Hospital of Chinese Medicine Affiliated to Nanjing University of Chinese Medicine, Nanjing, Jiangsu, China; 3 Institute of Modern Biology, Nanjing University, Nanjing, Jiangsu, China; 4 Science and Technology Experimental Center, Nanjing University of Chinese Medicine, Nanjing, Jiangsu, China

**Keywords:** SRSF2, proliferation, colorectal cancer, alternative splicing, AS, alternative splicing, CDK, cyclin-dependent kinases, CRC, colorectal cancer, EdU, 5-ethynyl-2′-deoxyuridine, RRM, RNA recognition motif, RS, serine/arginine-rich domain, siRNAs, small-interfering RNA, SR proteins, serine/arginine-rich proteins, SRSF2, serine/arginine-rich splicing factor 2, TCGA, The Cancer Genome Atlas, qRT-PCR, quantitative reverse transcriptase PCR

## Abstract

Aberrant expression of serine/arginine-rich splicing factor 2 (SRSF2) can lead to tumorigenesis, but its molecular mechanism in colorectal cancer is currently unknown. Herein, we found SRSF2 to be highly expressed in human colorectal cancer (CRC) samples compared with normal tissues. Both *in vitro* and *in vivo*, SRSF2 significantly accelerated the proliferation of colon cancer cells. Using RNA-seq, we screened and identified 33 alternative splicing events regulated by SRSF2. Knockdown of SLMAP-L or CETN3-S splice isoform could suppress the growth of colon cancer cells, predicting their role in malignant proliferation of colon cancer cells. Mechanistically, the *in vivo* crosslinking immunoprecipitation assay demonstrated the direct binding of the RNA recognition motif of SRSF2 protein to SLMAP and CETN3 pre-mRNAs. SRSF2 activated the inclusion of SLMAP alternative exon 24 by binding to constitutive exon 25, while SRSF2 facilitated the exclusion of CETN3 alternative exon 5 by binding to neighboring exon 6. Knockdown of SRSF2, its splicing targets SLMAP-L, or CETN3-S caused colon cancer cells to arrest in G1 phase of the cell cycle. Rescue of SLMAP-L or CETN3-S splice isoform in SRSF2 knockdown colon cancer cells could effectively reverse the inhibition of cell proliferation by SRSF2 knockdown through mediating cell cycle progression. Importantly, the percentage of SLMAP exon 24 inclusion increased and CETN3 exon 5 inclusion decreased in CRC samples compared to paired normal samples. Collectively, our findings identify that SRSF2 dysregulates colorectal carcinoma proliferation at the molecular level of splicing regulation and reveal potential splicing targets in CRC patients.

Dysregulation of alternative splicing (AS) has been demonstrated to play crucial roles in multiple types of tumors, which are triggered by abnormal alterations of splicing factors (1, 2, 3). As one kind of transacting factor, splicing factors identify and bind with *cis*-elements such as intronic and exonic enhancers or silencers to determine the reservation or removal of adjacent splice sites (4, 5). Aberrant expression or mutations of splicing factors fail to recognize the splice sites, thereby the alternative exons were included or excluded (4, 6). Multiple gene variants are generated by the regulation of AS and subsequently translated to differential proteins that participated in malignant proliferation, metastasis, or other progressions in carcinogenesis (7, 8, 9, 10).

Serine/arginine-rich proteins (SR proteins) are well-studied splicing factors that play crucial roles in splicing regulation. As one kind of RNA-binding protein, serine/arginine-rich splicing factor 2 (SRSF2) contains one RNA recognition motif (RRM) and one serine/arginine-rich domain (RS) (11). SRSF2 is involved in multiple molecular mechanisms both as a general splicing activator and a potential transcription activator (8, 12, 13). In addition, SRSF2 also participates in mRNA transport, mRNA translation, genome stability, and other molecular functions (14, 15, 16, 17). Importantly, many cancer-specific splice variants are dysregulated by the changes in SRSF2 expression (8, 18, 19, 20). Heterozygous mutations in SRSF2 occur in patients with myelodysplastic syndromes, chronic myelomonocytic leukemia, and myeloproliferative neoplasm, which are related to poor prognosis. The mutations in SRSF2 aberrantly regulate the splicing of some vital hematopoietic regulators in the above diseases (21, 22, 23, 24).

The switch in cell cycle progression is one of the main pathways for cell proliferation. Cyclins and cyclin-dependent kinases (CDKs) are key regulatory molecules which are essential for cell transition from G1 to S phase in the cell cycle process (25, 26, 27). However, it remains unclear whether SRSF2 promotes proliferation of colon cancer cells *via* mediating cell cycle.

Colorectal cancer (CRC) is one of the most prevalent digestive tumors and is the third most common human cancer according to the statistics (28). However, the AS profile regulated by SRSF2 is obscure in CRC.

Here we analyzed the expression of SRSF2 and profiled AS events in CRC samples using RNA sequencing analysis. We further revealed the function of SLMAP-L and CETN3-S splice variants and their regulator SRSF2 in proliferation of colon cancer cells. Cancer-related splice variants in SLMAP and CETN3 were validated by regulation of SRSF2 at the molecular level. Our data provide evidence with clinical implications as candidate prognostic factors in CRC patients.

## Results

### Splicing factor SRSF2 is upregulated in CRC patients

**Figure 1:**
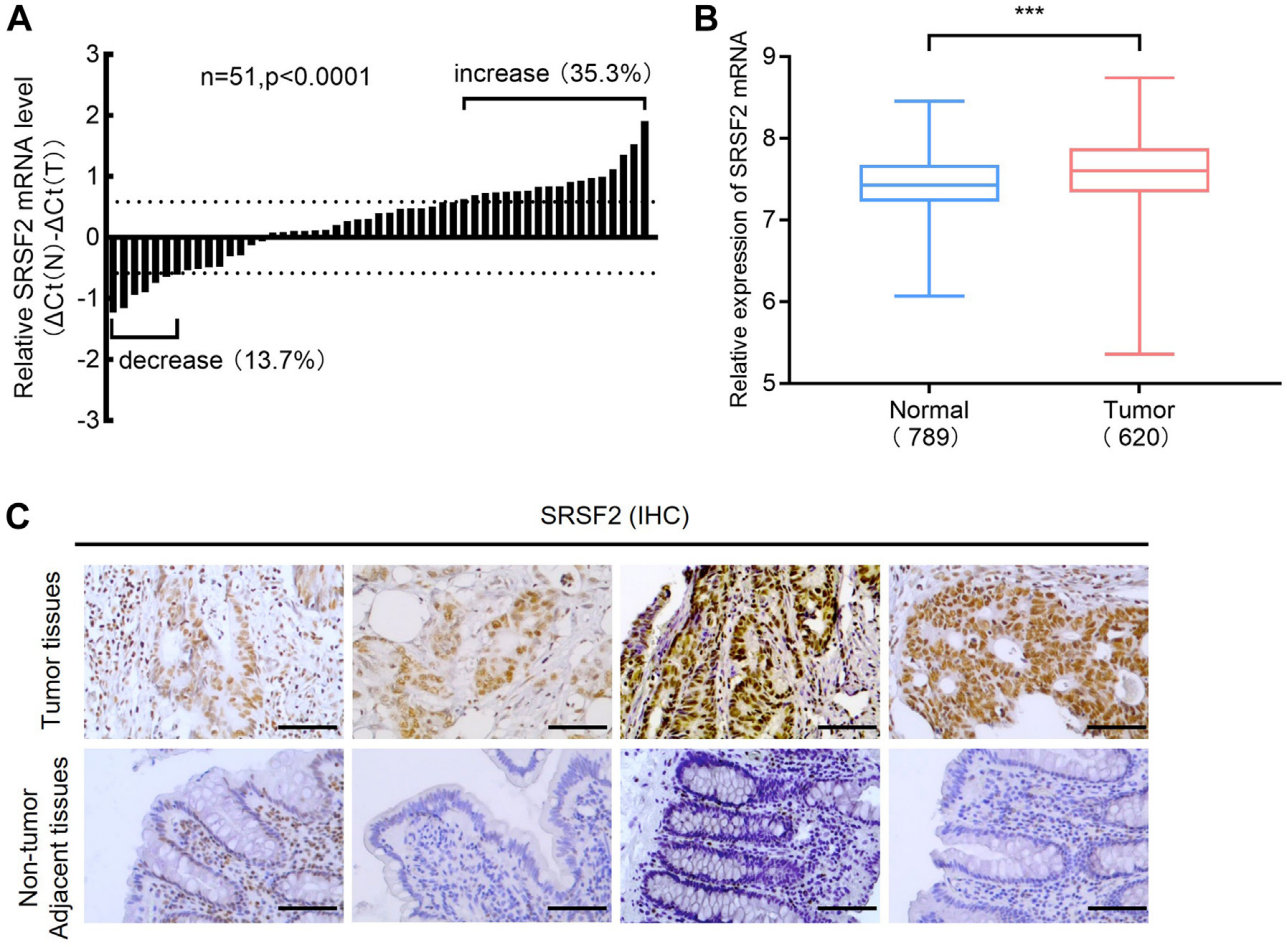
**SRSF2 is upregulated in human colorectal cancer samples.***A*, high expression of SRSF2 mRNA levels in CRC samples were revealed by qRT-PCR analysis. qRT-PCR was performed in 51 paired CRC RNA samples. SRSF2 mRNA expression levels were normalized to β-actin. The bar value is the log ratio of SRSF2 mRNA levels between CRC (T) and paired normal tissues (N). *p* < 0.0001. *B*, box plot compared SRSF2 mRNA levels in normal colorectal tissues (789 samples) and colorectal tumors (620 samples) in the published TCGA datasets and GTEx datasets. ∗∗∗*p* < 0.001. *C*, IHC staining of SRSF2 protein in representative CRC tumor tissues and adjacent nontumor tissues. Scale bar, 100 μm. CRC, colorectal cancer; qRT-PCR, quantitative reverse transcriptase PCR; SRSF2, serine/arginine-rich splicing factor 2; TCGA, The Cancer Genome Atlas.

To evaluate the expression profile of splicing factor SRSF2 in human CRC, we first tested mRNA levels of SRSF2 in 51 human CRC samples and their paired normal colorectal tissues by quantitative reverse transcriptase PCR (qRT-PCR). Upregulation of SRSF2 mRNA expression (>1.5-fold) occurred in 18 of 51 (35.3%) CRCs compared with normal colorectal tissues. Whereas only 7 of 51 (13.7%) CRCs had >1.5-fold decrease in SRSF2 expression compared to noncancerous samples (Fig. 1*A*). *In silico* analysis of the published datasets from The Cancer Genome Atlas (TCGA) (https://portal.gdc.cancer.gov/) and GTEx V8 version (https://gtexportal.org/home/datasets) including 620 CRC tissues (tumor) and 789 normal colorectal tissues (normal) revealed a significant increase of SRSF2 mRNA expression in CRC tissues compared with normal colorectal tissues (*p* < 0.001; Fig. 1*B*). SRSF2 IHC assays confirmed the increased protein expression of SRSF2 in CRC tumor tissues compared with the nontumor adjacent tissues (Fig. 1*C*). However, due to the limited number of human CRC samples that we collected from the hospital (Table S1) among which there were rare patients with tumor grade I or IV, we did not observe a significant relationship between SRSF2 mRNA levels and clinicopathological features (Table 1).

**Table 1:** Relationship between SRSF2 expression level and clinicopathological features in 51 patients with CRC

Clinical characteristics	Nonincreased SRSF2(n = 14)		Increased SRSF2(n = 37)		Test of significance
Gender	Number	%	Number	%	
Male	9	30.0%	21	70.0%	**C**^**2**^**= 0.2377**
Female	5	23.8%	16	76.2%	***p* = 0.6259**
Age					
>50	12	27.3%	32	72.7%	
≤50	2	28.6%	5	71.4%	***p* = 1**
Tumor site					
Colon	8	30.8%	18	69.2%	**C**^**2**^**= 0.2932**
Rectum	6	24.0%	19	76.0%	***p* = 0.5881**
T stage					
T1	1	50.0%	1	50.0%	
T2	2	22.2%	7	77.8%	
T3	7	23.3%	23	76.7%	
T4	4	40.0%	6	60.0%	***p* = 0.5494**
N stage					
N0	9	30.0%	21	70.0%	
N1	4	33.3%	8	66.7%	
N2	1	11.1%	8	88.9%	***p* = 0.5659**
M stage					
M0	14	28.6%	35	71.4%	
M1	0	0.0%	2	100.0%	***p* = 1.0000**
Tumor grade					
I	2	22.2%	7	77.8%	
II	7	33.3%	14	66.7%	
III	5	25.0%	15	75.0%	
IV	0	0.0%	1	100.0%	***p* = 0.8355**

Using SAS V8 statistical software, a Chi-square test (c^2^) or Fisher’s exact test was performed to analyze the relationship between SRSF2 mRNA levels with clinicopathological parameters. *p* < 0.05 was considered statistically significant and are indicated in bold. Tumor grade was based on histologic grade.

Abbreviation: TNM: tumor/node/metastasis.

To further study whether there are mutations of SRSF2 mRNA in clinical samples, we delved into the somatic mutations in CRC samples from the TCGA datasets. The top ten genes including *APC*, *TP53*, *TTN*, and *KRAS* are highly mutated in 556 CRC samples. However, there are rare mutations of SRSF2 in CRC samples (Fig. S1*A*). Only two patients had SRSF2 mutation, and the somatic mutation rate is only 0.36% in CRC samples, and the mutation sites are D48 G and S187Y (Fig. S1*B*).

Together, these observations strongly indicate that SRSF2 is increased in human colorectal cancer samples and predicted as a potential biomarker for CRC patients. Rare potential mutations of SRSF2 occurred in CRC samples.

### SRSF2 promotes proliferation of colon cancer cells

**Figure 2:**
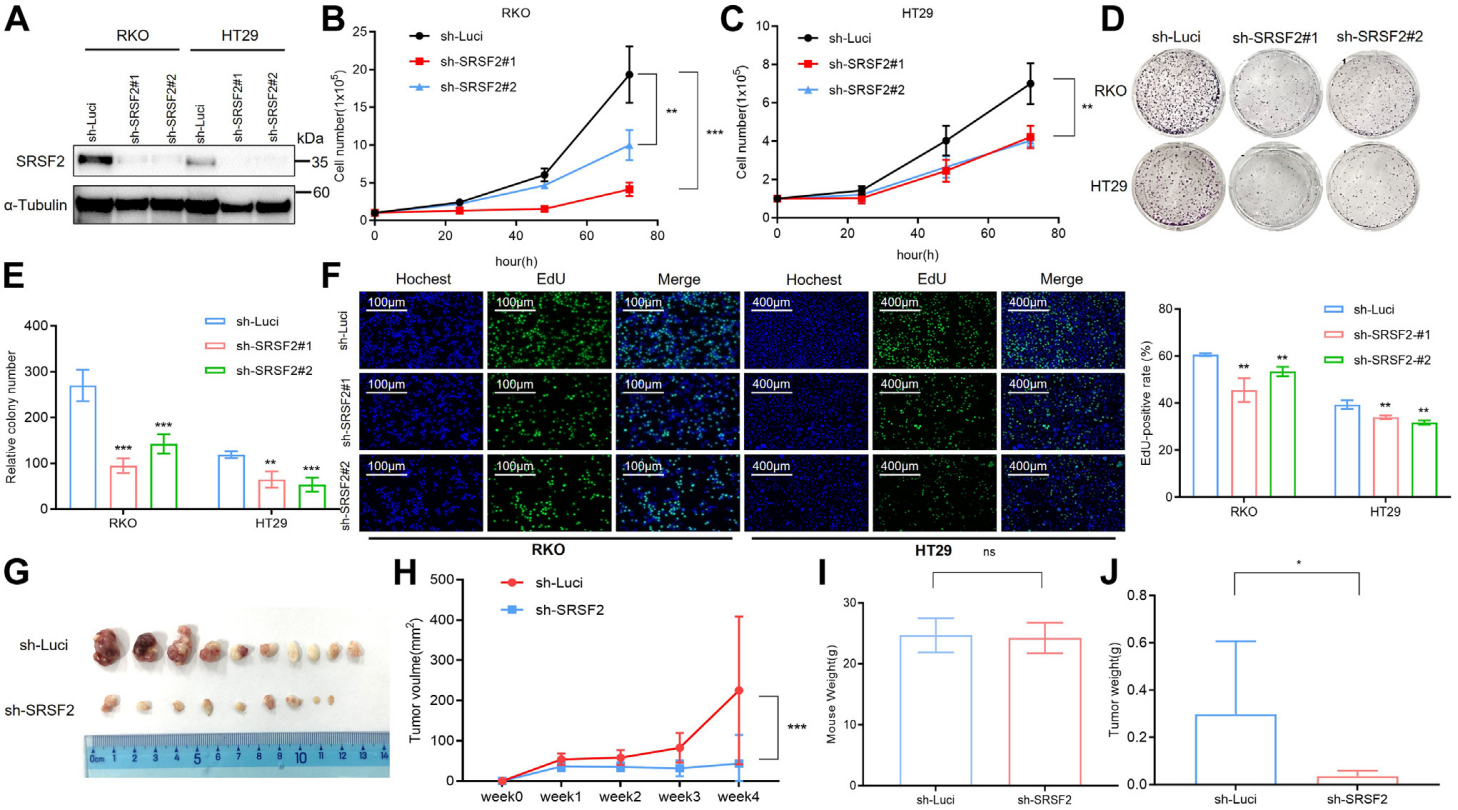
**Knockdown of SRSF2 inhibits the proliferation of colon cancer cells *in vitro* and *in vivo*.***A*, colon cancer cell lines RKO or HT29 cells were stably knockdown using lentiviruses transfected with SRSF2 shRNA (sh-SRSF2#1, sh-SRSF2#2) or negative control shRNA (sh-Luci). SRSF2 knockdown efficiency was assessed by Western blot. *B* and *C*, growth curve of RKO (*B*) or HT29 (*C*) cells after stably transfected with indicated shRNAs. ∗∗*p* < 0.01, ∗∗∗*p* < 0.001. *D* and *E*, clonogenic survival assay was performed, and crystal violet staining with representative cells were shown (*D*). The relative number of focal adhesions described in (*D*) was quantified as mean ± SD in the bar graph (*E*). ∗∗*p* < 0.01, ∗∗∗*p* < 0.001. *F*, the representative images of EdU staining assay for cells described in (*A*) were shown (*Left*). The results were presented as mean ± SD (*Right*). ∗∗*p* < 0.01. *G* and *H*, RKO cells stably expressing sh-SRSF2 and sh-Luci were injected into nude mice. Four weeks after injection, tumors excised from the mice were shown (*G*). Tumor volumes were measured every week, and time course of xenograft tumors growth was shown (*H*). ∗∗∗*p* < 0.001. *I*, mice weights. Results are shown as mean ± SD of mice weights (each with five mice). ns: no significance. *J*, weight of tumors excised from the mice. Results are shown as mean ± SD of tumor weights (each with initial ten injections). ∗*p* < 0.05. EdU, 5-ethynyl-2′-deoxyuridine; SRSF2, serine/arginine-rich splicing factor 2.

To investigate whether SRSF2 played vital roles in human colon cancer cells, we first examined the expression levels of SRSF2 in CRC cell lines and normal colon cells. Higher levels of SRSF2 protein were verified in CRC cell lines when compared with normal colon cells *via* Western blot (Fig. S2*A*). Then two independent shRNAs targeting against SRSF2 (sh-SRSF2#1 and sh-SRSF2#2) and control shRNA (sh-Luci) were designed and synthesized. RKO or HT29 cells were stably knockdown with these shRNAs, and the effects of SRSF2 knockdown were confirmed by Western blot analysis (Fig. 2*A*). The knockdown of SRSF2 significantly inhibited the growth of both RKO and HT29 cells compared with the cells treated with control shRNA (Fig. 2, *B* and *C*). Strikingly, SRSF2 knockdown also induced severe inhibition of colony formation of both RKO and HT29 colon cancer cells *in vitro* (Fig. 2, *D* and *E*). Moreover, a significant reduction of the known cell proliferating marker 5-ethynyl-2′-deoxyuridine (EdU) was observed in both colon cancer cells’ knockdown of SRSF2 in comparison with the control cells (Fig. 2*F*). Cell Counting Kit-8 assays verified that SRSF2 knockdown severely inhibited the growth of colon cancer cells (Fig. S2*B*). However, SRSF2 knockdown did not affect cell death nor apoptosis using Trypan blue staining and cell apoptosis analysis, respectively (Fig. S2, *C*–*E*). Overexpression of SRSF2 was performed in RKO cells, and the overexpression effect was confirmed by Western blot (Fig. S3*A*). Significantly, higher expression of SRSF2 promoted both colony formation of RKO cells and the proliferating marker EdU (Fig. S3, *B*–*D*). These observations indicated that the knockdown of SRSF2 inhibited the proliferation of colon cancer cells, and overexpression of SRSF2 promoted the proliferation of colon cancer cells *in vitro*.

To further validate the tumorigenic potential of SRSF2, we constructed the *in vivo* xenograft models. The RKO cells with stable knockdown of SRSF2 or the control cells were injected into nude mice independently. Compared with the control group, we observed a significant decrease in both tumor growth rate and tumor weight in SRSF2 knockdown groups, while there was no significance in the body weight between the two groups of mice (Fig. 2, *G*–*J*). Taken together, these *in vitro* and *in vivo* data confirmed that SRSF2 promoted proliferation in colon cancer cells.

### Landscape of SRSF2-affected AS events in RKO cells

**Figure 3:**
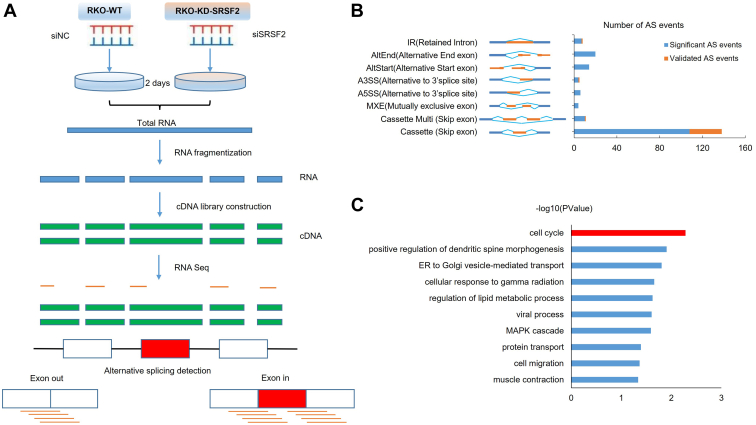
**Alternative splicing profiles affected by SRSF2 in RKO cells.***A*, RNA-seq strategy was performed using RKO cells transiently transfected with siSRSF2 or siNC independently. *B*, quantification of SRSF2-affected differential alternative splicing events, as revealed by analysis of RNA-seq data. Among these events, there are 108 cassette, 6 A5SS, 4 A3SS, 4 MEX, 7 IR, 10 Cassette Multi, 14 AltStart, and 20 AltEnd alternative splicing events. *C*, gene ontology analysis of SRSF2-targeted 173 splicing events. AS, alternative splicing; SRSF2, serine/arginine-rich splicing factor 2.

As one of the splicing factors, it is well proved that SRSF2 plays a critical role in splicing regulation. We conducted high throughout sequencing of RNA (RNA-seq) on the wildtype (WT) and SRSF2 knockdown (KD) RKO cells to profile the global AS events that might be regulated. Total RNA from si-NC and si-SRSF2 of RKO cells were extracted and the cDNA libraries were constructed after fragmentization. AS events were screened and detected after sequencing by HiSeq X (Illumina) on a 150 bp paired-end run (Fig. 3*A*). A total of 55,002,320 (WT, 96.4%) and 41,118,515 (SRSF2-KD, 96.4%) mapped reads were respectively generated after proper optimization. The rates of the unique mapped reads aligned to the reference genome in WT and SRSF2-KD cells were 88.8% and 88.5%, respectively, which indicated a high quality of the sequencing (Table 2). We found that SRSF2 knockdown affected 173 significant AS events (FDR < 0.05), including 108 cassette events (skip exon), 20 AltEnd (alternative end exon), 14 AltStart (alternative start exon), 10 Cassette_multi (skip exon), 6 alternative 5′ A5SS (alternative 5′ splice sites), 4 alternative 3′ A3SS (alternative 3′ splice sites), 4 MXE (mutually exclusive exons), and 7 IR (retained introns). A total of 47 AS events were screened and validated, among which isoform switches were significantly activated in 33 events (Table S2) containing 30 cassette exons, 1 Cassette_multi, 1 A3S, and 1 IR. Apparently, cassette events were the most affected events (Fig. 3*B*). Furthermore, the SRSF2-affected splicing targets were associated with tumor-related functions, such as cell cycle, cell migration, MAPK cascade, and ER to Golgi vesicle-mediated transport (Fig. 3*C*). Overall, SRSF2 is involved in all common modes of AS, among which cassette exons are the most frequent targets of SRSF2 regulation in colon cancer cells. And the functions of SRSF2-affected AS targets were related to tumorigenesis.

**Table 2:** Summary of RNA-seq data mapping results

#Item	NC-RKO	KD2-RKO
All	57,053,716	42,672,548
UnMapped	2,051,396	1,554,033
Mapped	55,002,320	41,118,515
MappedRate	96.40%	96.40%
UniqueMapped	50,652,092	37,774,260
UniqueMappedRate	88.80%	88.50%
RepeatMapped	4,350,228	3,344,255
JunctionAllMapped	19,244,046	14,035,884
JunctionUniqueMapped	17,083,660	12,413,266
AllBase	7,362,216,053	5,488,358,942
UnMappedBase	263,988,966	199,549,846
MappedBase	7,098,227,087	5,288,809,096
UniqueMappedBase	6,539,326,266	4,860,759,741
RepeatMappedBase	558,900,821	428,049,355

Unique mapped reads include paired-end reads with an unalignable mate. Repeat mapped reads have multiple alignments. All junction unique mapped reads have three or more offset alignment.

### Validation of SRSF2-dependent AS events in RKO cells

**Figure 4:**
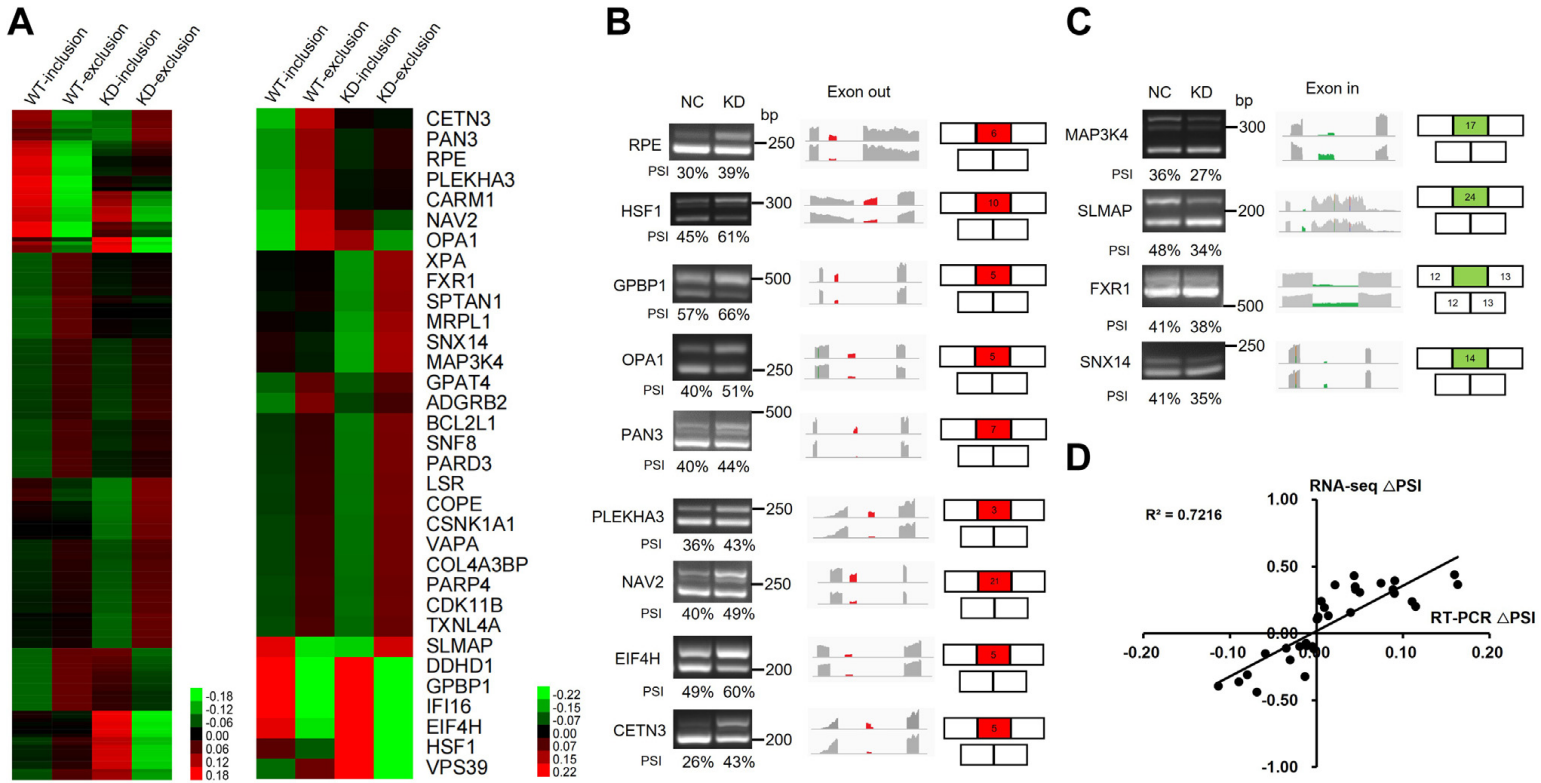
**Validation of SRSF2-regulated differential alternative splicing events in RKO cells.***A*, heatmap of the significant splicing profiling data by RNA-seq among the RKO/siNC (WT) and RKO/siSRSF2 (KD) groups (FDR < 0.05, *Left*), and the heatmap of the validated AS events were shown (*Right*). The data were sorted by the mean value of the indicated WT and KD groups analyzed. *Green* indicated *inclusion*, and *red* indicated *exclusion*. *B* and *C*, representative exon out events regulated by SRSF2 with RT-PCR results, RNA-seq reads coverage, and quantification of the RNA products measured as PSI (percent of splicing in). Note that alternative exons for SRSF2-mediated exclusion were marked in *red* (*B*). Representative exon in events affected by SRSF2 were shown and the alternative exons for inclusion were marked in *green* (*C*). *D*, correlation between ΔPSI from RNA-seq AS analysis and 33 RT-PCR validated splicing events. AS, alternative splicing; SRSF2, serine/arginine-rich splicing factor 2.

To validate SRSF2-dependent AS events in RKO cells, RT-PCR and IGV analysis were performed on these target genes to confirm the splicing pattern of alternative exons for SRSF2 regulation. The AS events affected by SRSF2, and the validated events were involved in the significant inclusion or exclusion of alternative exons (Fig. 4*A*). The representative 13 validated AS events were shown in Figure 4, *B* and *C*. SRSF2 knockdown could change the splicing pattern that either activated the inclusion of the alternative exon in mRNA variants such as RPE, HSF1, GPBP1, OPA1, PAN3, PLEKHA3, NAV2, EIF4H, and CETN3 (Fig. 4*B*) or repressed the inclusion of the alternative exon in mRNA variants such as MAP3K4, SLMAP, FXR1, and SNX14 (Fig. 4*C*). We compared the ΔPSI (PSI: percent of splicing in, △PSI = PSI_KD_ − PSI_NC_) between RT-PCR experimental validation and RNA-seq AS analysis and found they were highly correlated, reflecting the successful confirmation (Fig. 4*D*). Together, these endogenous mRNA transcripts were under the control of SRSF2 which could regulate the formation of both the exon-containing and exon-lacking isoforms.

### Knockdown of SLMAP-L or CETN3-S splice variant suppresses proliferation of human colon cancer cells

**Figure 5:**
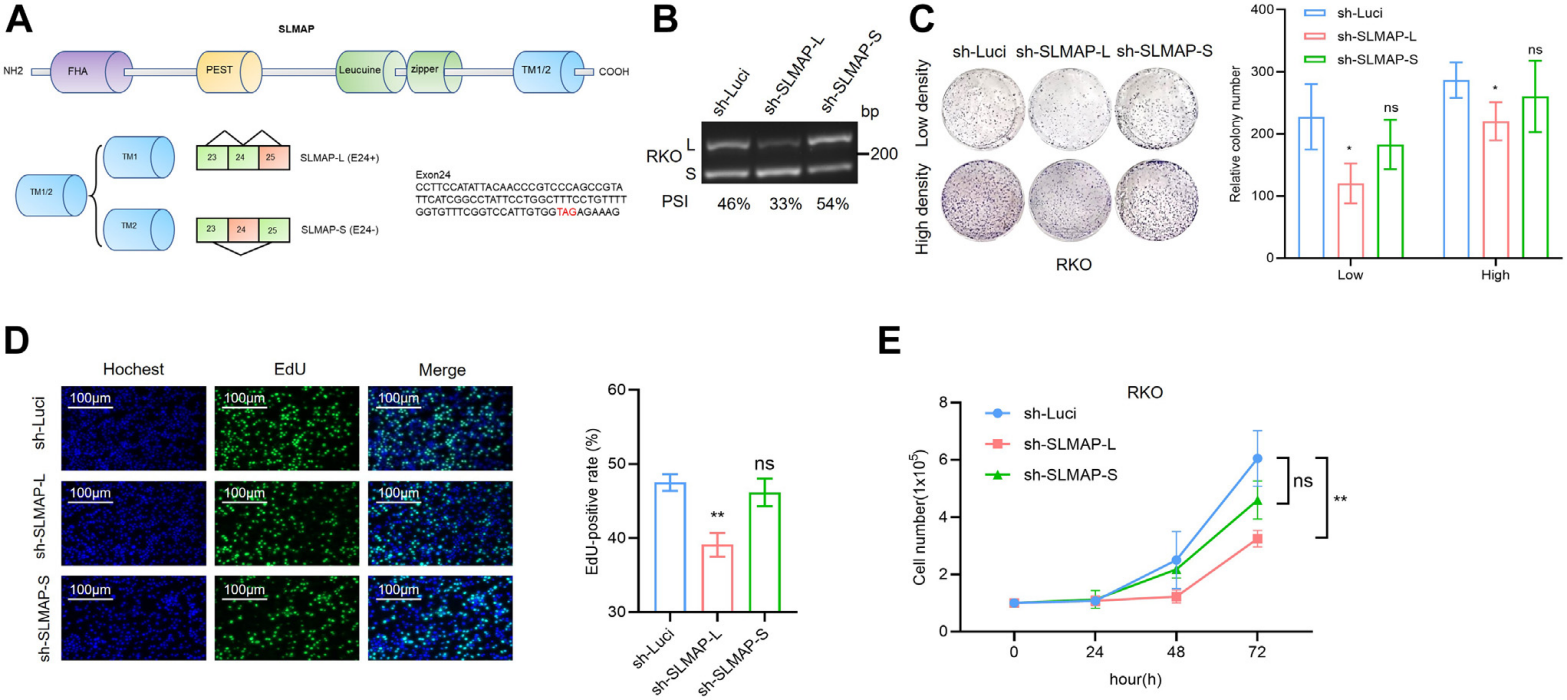
**Knockdown of SLMAP-L splice isoform inhibited coloncancercell growth *in vitro*.***A*, schematic diagram of SLMAP splice variants including or lacking alternative exon 24 (SLMAP-L and SLMAP-S). *B*, RKO cells were stably knockdown using lentiviruses transfected with isoform-specific shRNAs, which targeted against the SLMAP-L or SLMAP-S variants independently. SLMAP knockdown efficiency using sh-SLMAP-L or sh-SLMAP-S compared with sh-Luci in colon cancer RKO cells was assessed by RT-PCR analysis. The quantification of PSI was shown under the RT-PCR results. *C*, crystal violet staining with representative cells described in (*B*) in high and low density were shown after clonogenic survival assay performed (*Left*). The quantification of focal adhesions was shown as mean ± SD in the bar graph (*Right*). ns: no significance, ∗*p* < 0.05. *D*, the representative images of EdU staining assay for cells described in (*B*) were shown (*Left*). The results were presented as mean ± SD (*Right*). ns: no significance, ∗∗*p* < 0.01. *E*, growth curve of RKO cells after stably transfected with indicated shRNas. ns: no significance, ∗∗*p* < 0.01. EdU, 5-ethynyl-2′-deoxyuridine.

To identify the roles these validated AS events played in colon cancer cells and whether they were mediated by SRSF2, we first designed the specific shRNAs targeting the validated splice variants and detected the roles they played *in vitro*. SLMAP (sarcolemma associated protein) is one part of striatin-interacting phosphatase and kinases complexes. The inclusion of exon 24 introduces a premature termination codon in exon 24, causing the lack of exon 25 in the E24+ protein isoform which contains transmembrane domain TM1 domain in SLMAP. Whereas the exclusion of exon 24 (E24−) containing exon 23 and exon 25 will introduce another transmembrane domain TM2 domain in SLMAP. The splice isoform SLMAP-L represented the inclusion of exon 24 (E24+) and SLMAP-S represented the exclusion of exon 24 (E24−) (Fig. 5*A*). Similarly, the isoform including exon 5 (E5+) in CETN3 was defined as CETN3-L, and the isoform CETN3-S was with the exclusion of exon 5 (E5−) (Fig. S4*A*).

To investigate whether these splice variants were involved in the progression of colon cancer cells, the shRNAs respectively targeting against SLMAP-L (sh-SLMAP-L), SLMAP-S (sh-SLMAP-S), and CETN3-S (sh-CETN3-S), CETN3-L (sh-CETN3-L) were synthesized respectively. SLMAP-L, SLMAP-S, CETN3-S, or CETN3-L splice variant was stably knockdown individually in RKO cells using these shRNAs, and the knockdown effects of SLMAP-L, SLMAP-S, CETN3-S, and CETN3-L were confirmed by RT-PCR analysis (Figs. 5*B* and S4*B*). The cell growth of RKO cells was significantly repressed after the knockdown of SLMAP-L or CETN3-S, whereas the knockdown of SLMAP-S or CETN3-L had weak effects on RKO cells proliferation (Figs. 5, *C*–*E* and S4, *C*–*F*). SLMAP-L or SLMAP-S was overexpressed in RKO cells respectively, and the overexpression effect was verified by RT-PCR (Fig. S5*A*). Overexpression of SLMAP-L was observed to promote the proliferation of RKO cells; however, SLMAP-S overexpression had no significant effect on the growth of RKO cells (Fig. S5, *B*–*D*). In summary, SLMAP-L and CETN3-S splice variants were identified and proved to play crucial roles in the malignant proliferation of colon cancer cells *in vitro*.

### Insights into splicing mechanism of cancer-related splice variants by SRSF2 regulation

**Figure 6:**
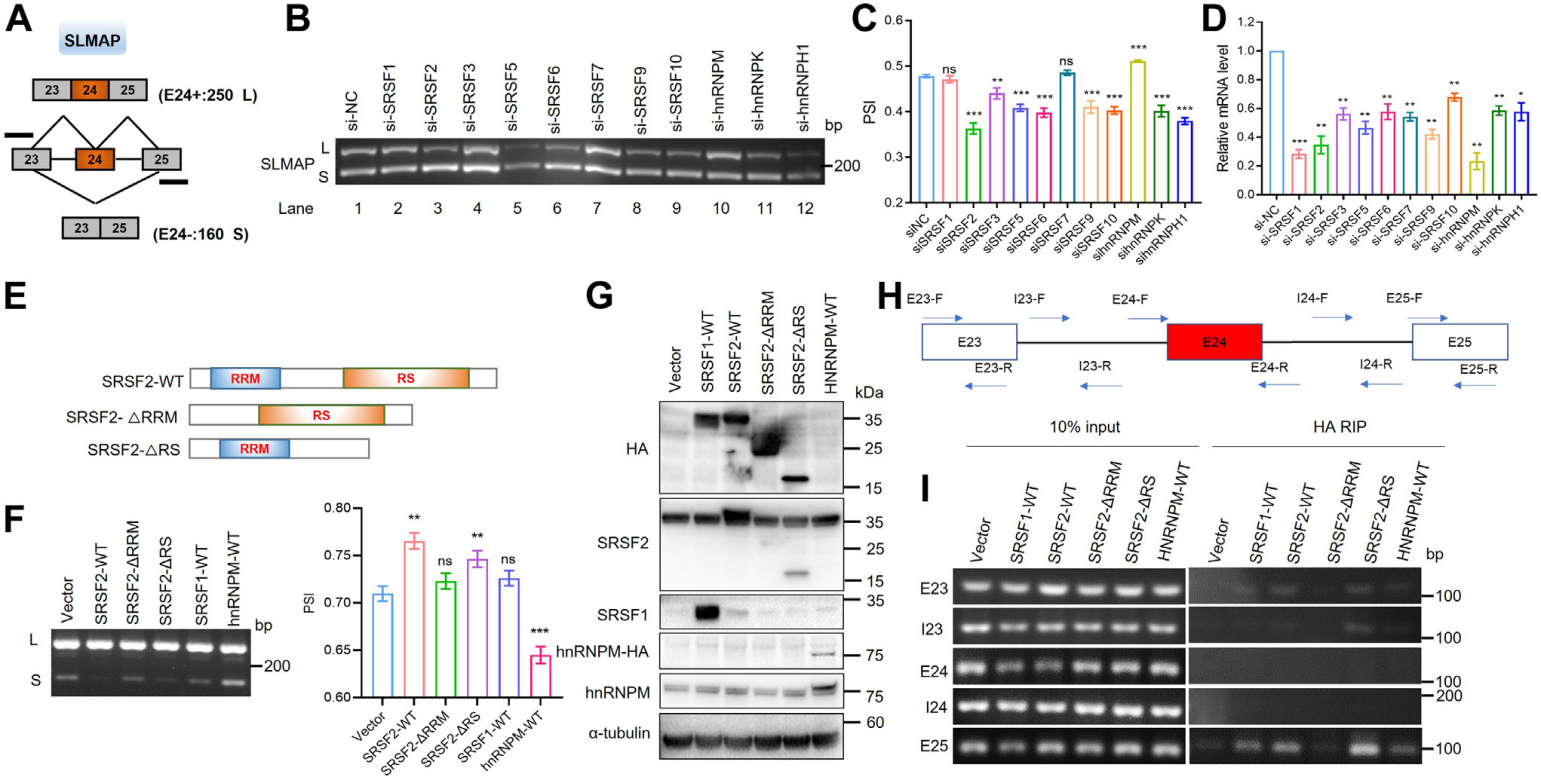
**SLMAP exon 24 is subject to the regulation of multiple splicing regulators including SRSF2 and binds with the RRM domain of SRSF2.***A*, diagrams for detection of SLMAP variants including or lacking alternative exon 24 (E24+ or E24−). The product sizes for the two variants of SLMAP are shown. *B* and *C*, the indicated siRNAs were transiently transfected into RKO cells, and the RNAs were extracted for RT-PCR analysis of SLMAP exon 24 inclusion/skipping (*B*). Each PSI value quantification from RT-PCR results of SLMAP splice variants was shown as mean ± SD in the bar graph (*C*). ns: no significance, ∗∗*p* < 0.01, ∗∗∗*p* < 0.001. *D*, the knockdown efficiency of indicated SR or hnRNP proteins using siRNAs compared with si-NC in RKO cells were assessed by qRT-PCR analysis. ∗*p* < 0.05, ∗∗*p* < 0.01, ∗∗∗*p* < 0.001. *E*, schematic diagram of RRM and RS domains in SRSF2, and constructions of two SRSF2 mutants: ΔRRM (deleting RRM domain), ΔRS (deleting RS domain). Both the mutants and SRSF2-WT plasmids were HA tagged. *F*, the indicated plasmids were transiently transfected into RKO cells, and RT-PCR was performed to analyze the inclusion/skipping of SLMAP exon 24. Each PSI value quantification from RT-PCR results was shown as mean ± SD in the bar graph. ns: no significance, ∗∗*p* < 0.01, ∗∗∗*p* < 0.001. *G*, Western blot of the endogenous SRSF1, SRSF2, hnRNPM with anti-SRSF1, anti-SRSF2, and anti-hnRNPM antibodies independently and the exogenous SRSF1, SRSF2, and hnRNPM with anti-HA antibodies. *H*, *upper*, diagram for the specific primers designed according to exon-intron (E-I) boundary sequences to detect exons 23 to 25 and introns 23 and 24. *Bottom*, RT-PCR analysis following CLIP assay indicated the direct binding between indicated proteins and endogenous SLMAP RNA fragments. CLIP, crosslinking immunoprecipitation; RRM, RNA recognition motif; RS, serine/arginine-rich domain; SRSF2, serine/arginine-rich splicing factor 2.

Human SLMAP and CETN3 pre-mRNAs are subject to AS regulation, thus long (L) and short (S) variants were generated (Figs. 6*A* and S4*A*), though the long variant of CETN3 was rarely expressed in untreated RKO cells (Lane 1 in Fig. S6*A*). To further explore the roles of splicing regulators involved in SLMAP and CETN3 splicing, the deletions of some SR and hnRNP proteins were performed using small-interfering RNA (siRNAs) in RKO cells. The specific primers were designed to detect alternative SLMAP exon 24 or CETN3 exon 5 individually by RT-PCR and the PSI (percent of splicing in) of each RNA product was quantified using Image J software (Figs. 6, *B* and *C* and S6, *A* and *B*). The knockdown effects were confirmed using qRT-PCR analysis (Fig. 6*D*).

In untreated RKO cells, the percent of exon 24 inclusion in SLMAP was about 47% (Lane 1 in Fig. 6, *B* and *C*). Deletion of hnRNPM increased exon 24 inclusion in SLMAP (Fig. 6*B*, compare lane 10 with lane 1, and Fig. 6*C*), while deletion of the other splicing regulators except for SRSF1 and SRSF7 repressed the inclusion of exon 24 among which SRSF2 deletion robustly repressed exon 24 inclusion (Fig. 6*B*, compare lane 3 with lane 1, and Fig. 6*C*). Furthermore, we found that the knockdown of SRSF1, SRSF2, SRSF5, SRSF6, or SRSF10 could increase exon 5 inclusion in CETN3 to more than 30% (Fig. S6*A*, compare lanes 2, 3, 5, 6 and 9 with lane 1, and Fig. S6*B*), among which SRSF2 deletion activated the inclusion of exon 5 to more than 40%. In contrast, knockdown of SRSF3, SRSF7, hnRNPM, or hnRNPK repressed the inclusion of exon 5 in CETN3 (Fig. S6*A*, compare lanes 4, 7, 10 and 11 with lane 1, and Fig. S6*B*). In total, SLMAP exon 24 or CETN3 exon 5 is subject to multiple splicing factors regulation, and the splicing factors could regulate different AS events in different ways.

SRSF2 contains one RRM at the N terminus and one arginine/serine domain (RS) at the C terminus. Based on the SRSF2-WT plasmid, we constructed the RRM deletion and RS deletion mutants, respectively (Fig. 6*E*). The overexpression of SRSF2 led to a significant increment of exon 24 inclusion in SLMAP, resulting in SLMAP-L isoform compared with the control group. The exogenous overexpression of the RS deletion mutant of SRSF2 had similar effects with SRSF2 WT overexpression in promoting SLMAP exon 24 inclusion. However, the RRM deletion mutant of SRSF2 had no effect on changes of exon 24 inclusion compared with the control group, indicating the crucial effects of SRSF2-RRM domain in regulating SLMAP exon 24 inclusion. Meanwhile, the overexpression of SRSF1 had no effects in regulating exon 24 splicing, hnRNPM could lead to the increment of exon 24 exclusion in SLMAP (Fig. 6*F*). Similarly, the SRSF2-RRM domain took an essential part in regulating the exclusion of CETN3 exon 5 (Fig. S6, *C* and *D*).

To confirm whether SRSF2 bind to SLMAP or CETN3 pre-mRNA, we transiently overexpressed HA-tagged SRSF2-WT, the RRM, or RS deletion mutants in 293T cells. We also transfected 293T cells using HA-Vector (Vector), HA-SRSF1-WT (SRSF1-WT), or HA-hnRNPM-WT (hnRNPM-WT) plasmids as controls to rule out the experimental artifacts caused by HA antigen (Fig. 6*G*). The *in vivo* crosslinking immunoprecipitation (CLIP) and RT-PCR analysis were carried out with specific primers for the indicated exons. SLMAP and CETN3 were the cases displayed as SRSF2-dependent exon inclusion and exon exclusion individually. Significantly, SRSF2-WT and RS deletion mutant (SRSF2-ΔRS) were observed to bind with exon 25 in SLMAP pre-mRNA and exon 6 in CETN3 pre-mRNA with high affinity. However, RRM deletion mutant (SRSF2-ΔRRM), SRSF1-WT, or hnRNPM-WT to both SLMAP exon 25 and CETN3 exon 6 were with low or even negligible affinities, similar to exon 23 or 24 in SLMAP and exon 4 or 5 in CETN3 (Figs. 6*H* and S6*E*). Indicating that SRSF2 binds to the pre-mRNA in SLMAP or CETN3 through the RRM domain. Both of the binding sites in pre-mRNAs of SLMAP and CETN3 were the flanking constitutive exons.

Based on the above results, multiple splicing factors were involved in SLMAP and CETN3 splicing, among which SRSF2 is the predominant splicing regulator through the binding of its RRM domain with pre-mRNA of SLMAP or CETN3.

### SRSF2 and its splicing targets SLMAP-L or CETN3-S regulate cell cycle progression in colon cancer cells

**Figure 7:**
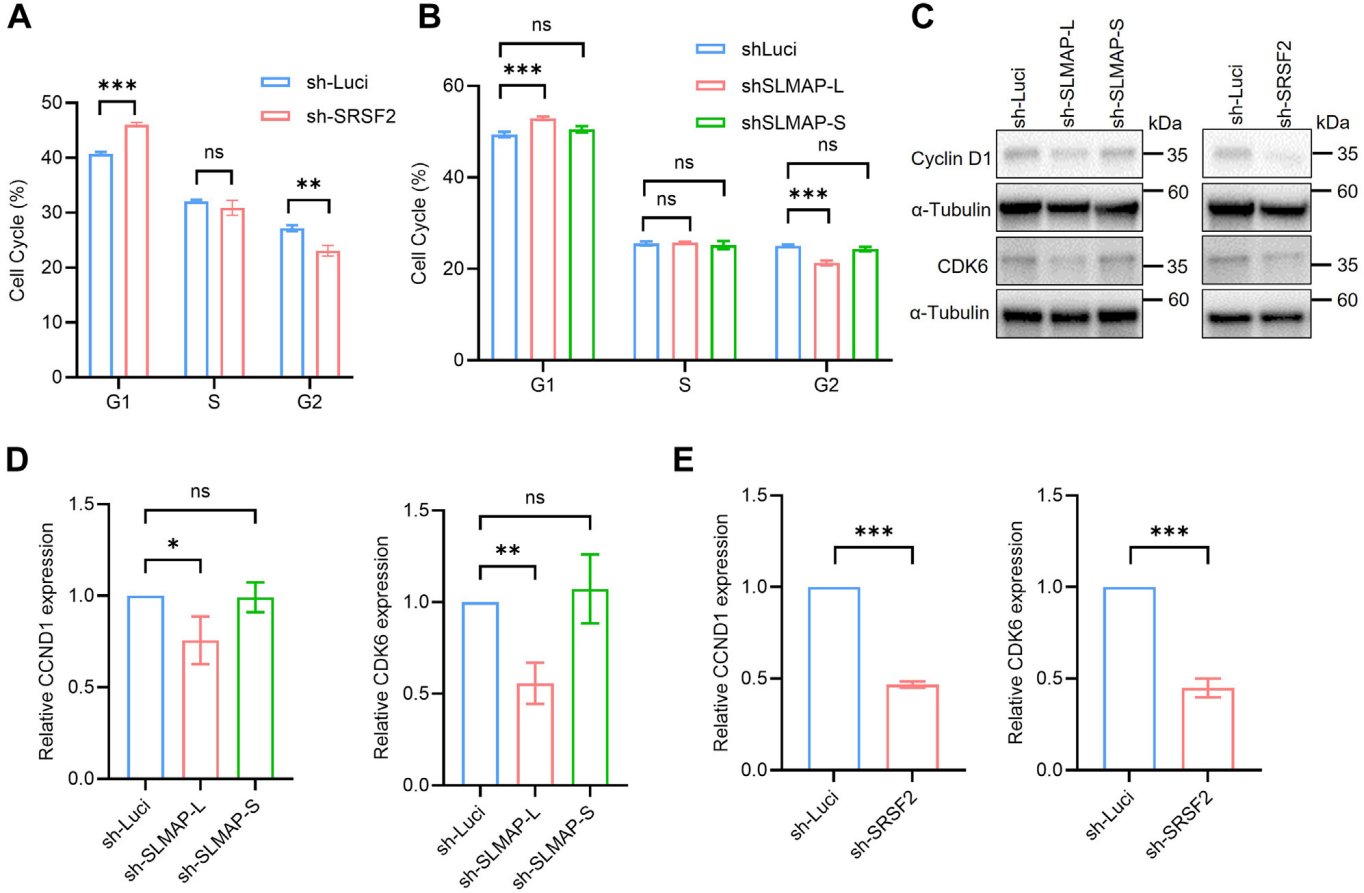
**Knockdown of SRSF2 or SLMAP-L induces G1 arrest in cell cycle progression.***A*, cell cycle was analyzed in SRSF2 knockdown RKO cells (sh-SRSF2) and the control RKO cells (sh-Luci). The quantification of the representative DNA content was shown as mean ± SD in the bar graph. ns: no significance, ∗∗*p* < 0.01, ∗∗∗*p* < 0.001. *B*, cell cycle was analyzed in SLMAP-L/SLMAP-S knockdown RKO cells (sh-SLMAP-L/sh-SLMAP-S) and the control RKO cells (sh-Luci). The quantification of representative DNA content was shown as mean ± SD in the bar graph. ns: no significance, ∗∗*p* < 0.01. *C*, Western blot of RKO cells knocking down SLMAP-L, SLMAP-S, SRSF2, or sh-Luci using anti-α-Tubulin, anti-Cyclin D1 and anti-CDK6 antibodies, independently. *D* and *E*, quantification of the Western blot described in (*C*). α-Tubulin is used to normalize the results, the relative CCND1 (Cyclin D1), or CDK6 expression of control cells was set as 100%. The data represent three independent experiments, each value was shown as mean ± SD in the bar graph. ∗*p* < 0.05, ∗∗*p* < 0.01, ∗∗∗*p* < 0.001. SRSF2, serine/arginine-rich splicing factor 2.

To our knowledge, previous studies had reported that SRSF2 was involved in the control of cell cycle progression (19, 27, 29). The top prediction by gene ontology analysis in our study showed that the splicing targets affected by SRSF2 were related to the cell cycle process (Fig. 3*C*). Cell cycle analysis deduced a significant G1 arrest after SRSF2 knockdown (Figs. 7*A* and S7*A*). SLMAP-L or CETN3-S knockdown induced a likewise G1 arrest as SRSF2 knockdown, while SLMAP-S or CETN3-L knockdown had no significant effects on cell cycle progression (Figs. 7*B*, S4*G* and S7*B*). Since complex 1 formed by Cyclin D1 and CDK6 are necessary for cell transition from G1 to S phase in the cell cycle (27), Western blot analysis was conducted and verified that either SLMAP-L or SRSF2 knockdown decreased Cyclin D1 or CDK6 protein levels, while SLMAP-S knockdown did not have effects on Cyclin D1 or CDK6 levels (Fig. 7, *C*–*E*). Likewise, CETN3-S knockdown decreased Cyclin D1 levels, while CETN3-L knockdown had no effects on Cyclin levels (Fig. S4, *H* and *I*). The Western blot analysis kept consistent with cell cycle analysis. Therefore, we confirmed that SLMAP-L or CETN3-S promotes the proliferation of colon cancer cells like SRFS2 by facilitating cell cycle progression.

### Restoration of SLMAP-L or CETN3-S partially reverses the anti-proliferation effects in SRSF2-knockdown colon cancer cells by mediating cell cycle progression

**Figure 8:**
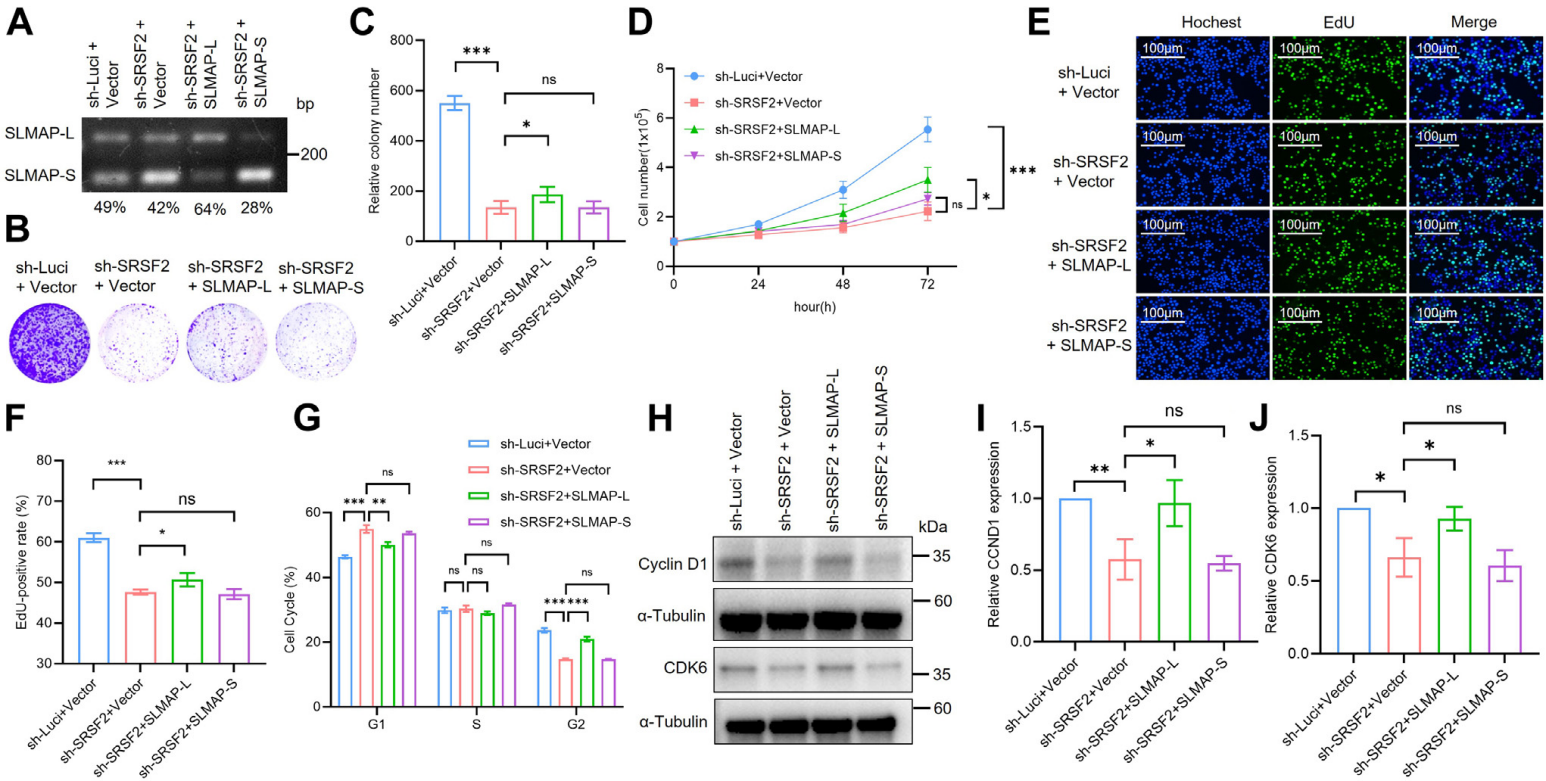
**Restoration of SLMAP-L reverses the inhibiting effects of SRSF2 knockdown on RKO cell proliferation.***A*, RT-PCR detection was performed to analyze the inclusion/skipping of SLMAP exon 24 in SRSF2 knockdown RKO cells stably transfected with SLMAP-L or SLMAP-S. Each PSI value quantification from RT-PCR results was shown under the PCR image. *B* and *C*, clonogenic survival assay for cells described in (*A*) was performed, and crystal violet staining with representative cells were shown (*B*). The relative number of focal adhesions described in (*B*) was quantified as mean ± SD in the bar graph (*C*). ns: no significance, ∗*p* < 0.05, ∗∗∗*p* < 0.001. *D*, growth curve of RKO cells described in (*A*) was shown. ns: no significance, ∗*p* < 0.05, ∗∗∗*p* < 0.001. *E* and *F*, the representative images of EdU staining assay for cells described in (*A*) were shown (*E*). The results were presented as mean ± SD (*F*). ns: no significance, ∗*p* < 0.05, ∗∗*p* < 0.01. *G*, cell cycle was analyzed in cells described in (*A*). The quantification of representative DNA content was shown as mean ± SD in the bar Graph. ns: no significance, ∗∗*p* < 0.01, ∗∗∗*p* < 0.001. *H*, Western blot of cells described in (*A*) using anti-α-tubulin, anti-Cyclin D1, and anti-CDK6 antibodies independently. *I* and *J*, quantification of the Western blot was shown described in (*H*). α-Tubulin is used to normalize the results, the relative CCND1 (*I*) or CDK6 (*J*) expression of control cells was set as 100%. The data represent three independent experiments, each value was shown as mean ± SD in the bar Graph. ns: no significance, ∗*p* < 0.05, ∗∗*p* < 0.01. EdU, 5-ethynyl-2′-deoxyuridine; SRSF2, serine/arginine-rich splicing factor 2.

We next decided to investigate whether SLMAP-L or CETN3-S splice isoform mediates the effects of SRSF2 on the proliferation of colon cancer cells. We stably introduced SLMAP-L, SLMAP-S, CETN3-L, or CETN3-S into sh-SRSF2 RKO cells respectively to overexpress the corresponding protein and infected the control plasmid into sh-Luci or sh-SRSF2 RKO cells. Overexpression of SLMAP-L/S or CETN3-L/S was verified by RT-PCR analysis (Figs. 8*A* and S8*A*). The restoration of SLMAP-L or CETN3-S but not SLMAP-S and CETN3-L, efficiently reversed the effects of SRSF2 knockdown on the proliferation of colon cancer cells (Figs. 8, *B*–*F* and S8, *B* and *C*).

To further explore whether SLMAP-L or CETN3-S mediates the function of SRSF2 in colon cancer cells *via* cell cycle progression, cell cycle assays were performed. In consistence with the results above, SRSF2 knockdown severely induced G1 arrest, while much lower G1 arrest was observed when complemented with SLMAP-L or CETN3-S isoform (Figs. 8*G*, S7*C* and S8*D*). Moreover, Western blot verified the restoration of SLMAP-L, but not the SLMAP-S splice isoform, which could significantly reverse the reductions in the expression of Cyclin D1 or CDK6 caused by SRSF2 knockdown (Fig. 8, *H*–*J*). CETN3-S also rescues the reduced expression of Cyclin D1, while CETN3-L could not rescue the reduced Cyclin D1 expression (Fig. S8, *E* and *F*). In summary, the restoration of SLMAP-L or CETN3-S splice isoform could reverse the suppressions of proliferation affected by SRSF2 knockdown by mediating the cell cycle progression in colon cancer cells.

### SLMAP exon 24 inclusion or CETN3 exon 5 exclusion increase in tumor samples of CRC patients

**Figure 9:**
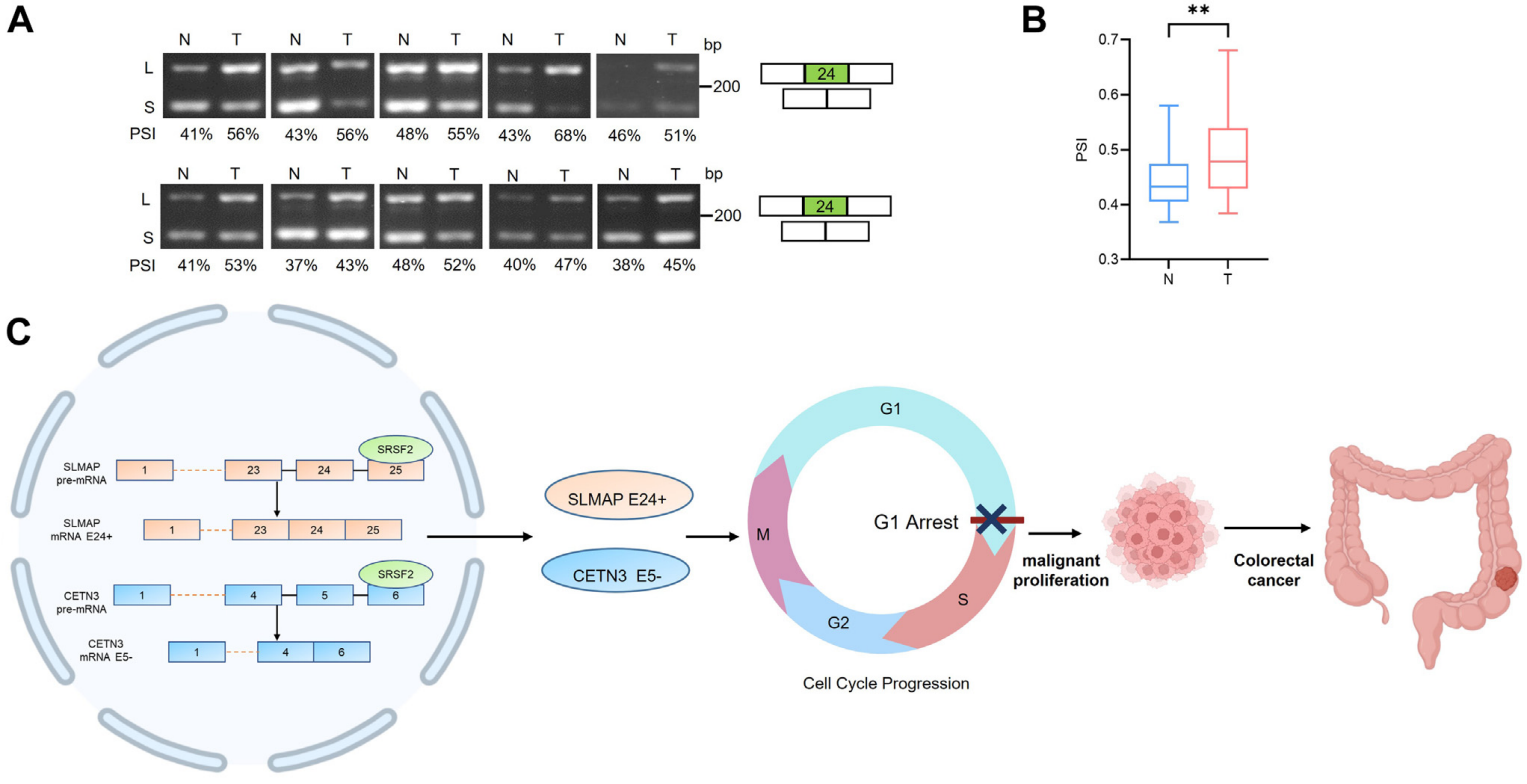
**The inclusion of SLMAP exon 24 increase in CRC tumor samples.***A* and *B*, representative RT-PCR results for splicing patterns of SLMAP transcripts are shown between colorectal tumors (T) and paired normal tissues (N), and the PSI quantification of each splice variant in SLMAP was shown under the RT-PCR results. Note that alternative exon 24 in SLMAP was marked in *green* (*A*). The PSI value quantification from RT-PCR results of SLMAP splice variants were presented as mean ± SD (*B*). ∗∗*p* < 0.01. *C*, schematic diagram of the proposed mechanism by which SRSF2 promotes malignant proliferation of CRC *via* cell cycle progression through regulating alternative splicing of SLMAP and CETN3 pre-mRNA. CRC, colorectal cancer; SRSF2, serine/arginine-rich splicing factor 2.

The expression patterns of several SRSF2-affected AS events were examined by RT-PCR analysis in 24 paired CRC samples with high SRSF2 mRNA expression. Although there were no significant splicing variations in splicing patterns of GPBP1, OPA1, EIF4H, and NAV2 examined in CRC samples compared with control tissues (data not shown), SLMAP and CETN3 were shown significant AS alterations independently. Of the 24 pairs of CRC samples tested, three pairs of SLMAP AS analysis and four pairs of CETN3 AS analysis were excluded for further statistical analysis because none of these samples had visible bands in the RT-PCR assays. Strikingly, increased inclusion of alternative exon 24 in SLMAP was observed in 17 of 21 CRC samples compared with normal tissues, and the representative images were shown (Fig. 9, *A* and *B*). Furthermore, increased exclusion of CETN3 exon 5 was detected in 14 of 20 CRC samples (Fig. S8*G*). In conclusion, the increment of SLMAP exon 24 inclusion and CETN3 exon 5 exclusion were observed in human colorectal cancer and predicted as potential targets for CRC patients.

## Discussion

In the present work, our data presented a comprehensive study of SRSF2 and its downstream AS profile in CRC. We first revealed that SRSF2 was significantly upregulated in clinical CRC samples. Multiple splicing events were identified in colon cancer cells through transcriptome analysis. SRSF2 regulated cancer-related SLMAP and CETN3 to generate different splice variants with distinct functions in cancer. SLMAP-L, CETN3-S, and their splicing regulator SRSF2 stimulated the proliferation of colon cancer cell. SRSF2, SLMAP-L, and CETN3-S splice variants were related to G1 arrest in the cell cycle progression. Rescue of SLMAP-L or CETN3-S in SRSF2-knockdown cells could partially reverse the antiproliferation effects *via* mediating cell cycle progression. These findings indicate that abnormal expression of SRSF2 regulates the expression of cancer-related splice variants and further contributes to colorectal cancer development.

As a well-characterized splicing regulator, SRSF2 regulated AS events in a highly position-dependent manner and has been implicated essential roles in vertebrate development and cancer progression (8, 12, 13, 30). Here, we demonstrated that SRSF2 controls many AS events by modulating both exon activation and repression in colon cancer cells through RNA-seq analysis and RT-PCR validation. CLIP assay revealed the direct binding between the RRM domain in SRSF2 protein and several pre-mRNAs. Moreover, we observed some other splicing factors also affected the AS of the above events, although they had lower affinity in binding with the pre-mRNAs of these splice variants. These findings confirm the possible cooperative or competitive link between SR and hnRNP proteins, reflecting the combinative regulation of AS factors (31).

Our data identified that SRSF2 was highly expressed in CRC tumors and promoted the proliferation of colon cancer cells *in vitro* and *in vivo*. Supportively, previous studies showed the essential roles of SRSF2 in cancer cell growth and survival (8). More importantly, we noted that SLMAP-L and CETN3-S splice variants shared similar proliferation-promoting functions in colon cancer cells. SLMAP encodes sarcolemma-associated protein, which is one of the main architectures of striatin-interacting phosphatase and kinases complexes that were involved in regulation of cell cycle, apoptosis, and cell migration (32). Two variants of SLMAP contained two distinct transmembrane domains TM1 and TM2 individually at C terminus and expressed in different subcellular structure. The variant containing TM1 domain includes exon 24 which introduces an inframe stop codon, while the other variant with TM2 domain excludes exon 24 (33, 34). A previous study showed that mutants of SLMAP that lack forkhead-associated domain repressed cell growth *via* cell cycle progression (32). CETN3 also named as centrin 3 which is a member of the centrin protein family and plays a fundamental role in centrosome duplication and separation (35). As shown in Figure 7 and Fig. S4, the cell cycle assay together with Western blot analysis revealed that the knockdown of SLMAP-L or CETN3-S led to G1 arrest in cell cycle progression. In this study, the elevated inclusion of SLMAP exon 24 and reduced inclusion of CETN3 exon 5 were observed in CRC tumor samples. Both SLMAP and CETN3 were subject to AS regulation of SRSF2. Moreover, our data demonstrated that SLMAP-L and CETN3-S splice isoforms played a proproliferation role in colon cancer progression and indicated to be potential therapeutic targets.

Moreover, previous studies showed the frequent mutations on P95 proline residue in SRSF2 occur in patients with myelodysplastic syndromes (36, 37). In this study, we predicted the top ten mutation events such as APC, TP53 and TTN frequently happened in CRC samples which were also reported previously (38, 39, 40). However, only 0.36% somatic mutation rate of SRSF2 occurred in CRC patients. This strongly indicated that abnormal high expression of SRSF2 but not the mutations in SRSF2 were the major factor in the progress of CRC development.

## Conclusion

In summary, we observed high expression levels of SRSF2 in CRC samples. Our data provided evidence that SRSF2 activated SLMAP-L splice isoform through binding with exon 25 and promoted CETN3-S splice isoform through binding with exon 6 (Fig. 9*C*). The knockdown of SRSF2 and its cancer-associated splicing events SLMAP-L or CETN3-S decreased malignant proliferation of colon cancer cells. SRSF2 and its splicing targets SLMAP-L or CETNS were involved in cell cycle progression. Restoration of SLMAP-L or CETN3-S partially abolishes the antiproliferation effects of SRSF2 knockdown *via* mediating cell cycle progression in colon cancer cells. The increasement of SLMAP-L and CETN3-S splice variants were identified in human colorectal cancer tumor samples compared with the paired normal samples. These findings identify that abnormal high expression of SRSF2 dysregulates the expression of cancer-related splice variants and revealed potential splice variants in patients with CRC.

## Experimental procedures

### Human CRC samples

Fifty-one pairs of clinical CRC samples and the corresponding control tissues were obtained and approved by the Ethics Committee of Nanjing Hospital of Chinese Medicine Affiliated to Nanjing University of Chinese Medicine (KY2021079). The fresh samples were collected without any treatment and placed in RNAlaterTM (Thermo Fisher Scientific) and stored at −80 °C for RNA expression detection and splicing events validation.

### Cell culture and reagents

Human CRC cell lines (RKO and HT29) were purchased from National Collection of Authenticated Cell Cultures (Shanghai Institute of Cell Biology, Chinese Academy of Sciences). Human embryonic kidney 293T cells were purchased from Beyotime Biotechnology. RKO and 293T cell lines were cultured in Dulbecco’s Modified Eagle Medium (Gibco) supplemented with 10% fetal bovine serum (BI, 04-004-1ACS), 100 U/ml penicillin, and 100 mg/ml streptomycin (Gibco). HT29 cells were cultured in McCoy’s 5A (Gibco), supplemented with 10% fetal bovine serum, 100 U/ml penicillin, and 100 mg/ml streptomycin. All cell lines were placed in a humidified incubator containing 5% CO_2_ at 37 °C.

### Cell growth, proliferation, and viability assays

Cell growth was quantified by directly counting the number of the cells or with Cell Counting Kit-8. Briefly, triplicate cells were separately seeded into a 12-wells plate, 1 × 10^5^ cells for each well and maintained for 24, 48, and 72 h. The proliferating cells was measured using EdU (Beyotime Biotechnology) staining (41). Colony formation assays were performed to examine cell survival. For details, 3000 cells were seeded in one well for 6-well plates and were cultured for 8 to 10 days in normal medium. The cell colonies were staining with crystal violet after fixing in 4% paraformaldehyde at room temperature. The colony numbers were determined from four random areas for each group. Cell viability was verified by counting the unstained cell numbers using a hemocytometer after trypan blue staining.

### siRNAs, shRNAs, and plasmids

The siRNA oligos targeting SRSF1, SRSF2, SRSF3, SRSF5, SRSF6, SRSF7, SRSF9, SRSF10, hnRNPM, hnRNPK, and hnRNPH1 were synthesized by Gene Pharma Company, the negative control si-NC was included. These siRNAs were transfected into cells with X-tremeGENE siRNA Transfection Reagent (ROCHE). The sequences were listed in Table S3.

The shRNAs specifically targeting SRSF2 (sh-SRSF2), SLMAP-Long isoform (sh-SLMAP-L), SLMAP-Short isoform (sh-SLMAP-S), CETN3-Long isoform (sh-CETN3-L), CETN3-Short isoform (sh-CETN3-S), and negative control (sh-Luci) were independently expressed in the knockdown lentiviruses vector PLKO.1-puro. The designed shRNAs were synthesized by Sangon Biotech. The shRNA sequences were listed in Table S4.

The wildtype SRSF2 (SRSF2-WT), SRSF2 domain deletion mutants (SRSF2-△RRM and SRSF2-△RS), the wildtype SRSF1 (SRSF1-WT), and wildtype hnRNPM (hnRNPM-WT) were constructed into pcDNA3.0 vector containing HA tag. Plasmids expressing SRSF2, SLMAP-L, SLMAP-S, CETN3-L, and CETN3-S transcript were constructed into PCDH-puro vector independently and synthesized by Sangon Biotech.

### Stable colon cancer sub-cell lines

The stable sub-cell lines from colon cancer RKO cell lines were constructed by infecting knockdown or expressing lentivirus expressing PLKO.1-puro or PCDH-puro individually which were selected with puromycin.

### *In vivo* xenograft tumor formation assay

The male BALB/c nude mice at the age of 5 weeks were purchased from Changzhou CAVENS Laboratory Animal Company. The stable RKO sub-cell lines expressing sh-Luci and sh-SRSF2, 3 × 10^6^ cells each group containing 0.25 v/v matrigel (Corning Incorporated) were subcutaneously injected at each dorsal flank into nude mice. Tumor size was measured every week using a vernier caliper. All nude mice were maintained in specific pathogen-free condition, and all animal experiments were approved by the Animal Ethics Committee of Nanjing University of Chinese Medicine (No. 202112A076).

### Reverse transcriptase PCR and quantitative reverse transcriptase PCR

The total RNAs from indicated cell lines were extracted using Trizol reagent (Beyotime Biotechnology). Reverse transcription from total RNAs were performed using M-MLV reverse transcriptase (Promega Corporation) and oligo-dT primers (Sangon Biotech) following the manufacturer’s instructions. The qRT-PCR was carried out using SYBR Green Master Mix (Yeason). For RT-PCR and qRT-PCR analysis, primers were designed and synthesized by Sangon Biotech and listed in Table S5.

### RNA sequencing and data analysis

RNA sequencing was performed by NovelBio. Total RNA samples were extracted from si-NC and si-SRSF2 of RKO cells. Each RNA sample was used to construct the cDNA libraries using the TruSeq Stranded mRNA Library Prep Kit (Illumina, Inc) according to the manufacturer’s instructions. The libraries were quality controlled with Agilent 2200 and sequenced by HiSeq X (Illumina) on a 150 bp paired-end run. Clean reads were obtained after the adaptor sequences, and low-quality reads were removed from the raw reads. Reads mapping was carried out after the clean reads obtained. Hisat2 was used to align the clean reads to human genome (GRCh38, Ensembl100) (42). Gene expression was determined by gene counts and RPKM method hTseq using hTseq (43). The raw data have been submitted to Gene Expression Omnibus with accession number GSE200272.

### IHC, Western blot, and *in vivo* CLIP

IHC was performed with anti-SC35 antibody (abcam, ab204916) as previously described (44). Western blot was carried out as previously described (45). The indicated primary antibodies used in this study were listed in Table S6. *In vivo* CLIP of SLMAP exon 25 or CETN3 exon 6 pre-mRNA bound to SRSF1, SRSF2, and hnRNPM proteins were performed as described previously (2). Briefly, hemagglutinin-tagged SRSF1, hnRNPM, SRSF2, and its domain deletion mutants or empty vector control were transfected into 293T cells transiently, and ultraviolet cross-linking was performed followed by immunoprecipitation using Magna RIP kit (Millipore).

### *In silico* analysis of SRSF2 expression and mutation

The mRNA expression levels of SRSF2 were analyzed using data from CRC samples in the TCGA database (https://portal.gdc.cancer.gov/) and GTEx V8 version (https://gtexportal.org/home/datasets). The mutation data were downloaded from TCGA database and visualized using the “maftools” package (https://bioconductor.org/packages/release/bioc/html/maftools) and analyzed in R software v4.0.3 (R Foundation for Statistical Computing), as described previously (46, 47).

### Cell cycle and apoptosis analysis

Cells used for cell cycle analysis were collected using 0.25% trypsin, centrifuged at 1000*g* for 5 min to remove trypsin, washed with 1 ml cold phosphate buffered saline (PBS), and re-suspended in 1 ml cold 70% ethyl alcohol. Then, cells were kept at 4 °C for 24 h and subsequently centrifuged at 1000*g* for 5 min, followed by washing with 1 ml cold PBS and centrifugation (1000*g*, 5 min). Cells were stained with 25 μl propidium iodide (C1052-2, Beyotime) and 10 μl rNase (C1052-3, Beyotime) at 37 °C for 30 min. The cell cycle was detected by the Gallios Flow Cytometer (A94303, Beckman).

Cells used for apoptosis analysis were harvested and prepared according to the manufacturer’s instructions (40302ES, YEASEN). Cells were collected using 0.25% trypsin, centrifuged at 300*g* for 5 min to remove trypsin, washed twice with 1 ml cold PBS, and centrifuged at 300*g* for 5 min to remove PBS. Then cells were resuspended in 100 μl 1× binding buffer. For each sample, 5 μl of Annexin-V–FITC, and 10 μl propidium iodide were added to the cell suspension and incubated for 15 min at room temperature (25 °C) in the dark. The stained cells were detected by the Gallios Flow Cytometer (A94303) and analyzed using FlowJo (Version 10.8.1).

### Statistical analysis

Statistical analysis was performed using Student’s *t* test. The PSI quantification from RT-PCR was done by ImageJ software. Statistical analyses of clinical CRC samples were performed using SAS software. Gene ontology analysis of SRSF2-affected splicing events were perform using Database for Annotation Visualization and Integrated Discovery (DAVID, david.ncifcrf.gov/) (48).

## Data availability

The data generated in this study are available in the article, tables, and supplementary files.

## Supporting information

This article contains supporting information.

## Conflict of interest

All the authors declare that they have no conflicts of interest with the contents of the article.
