# Supplementary material for: Comprehensive analysis of RNA-binding protein SRSF2-dependent alternative splicing signature in malignant proliferation of colorectal carcinoma
Source: J Biol Chem. 2023 Jan 6;299(2):102876. doi: 10.1016/j.jbc.2023.102876 (PMC9926302; doi:10.1016/j.jbc.2023.102876)
Supplement: Supplemental Figures S1–S8 and Tables S1–S6 [file mmc1.docx]

**Supplementary Figure and Figure Legend**

**Supplementary Figure 1**

**
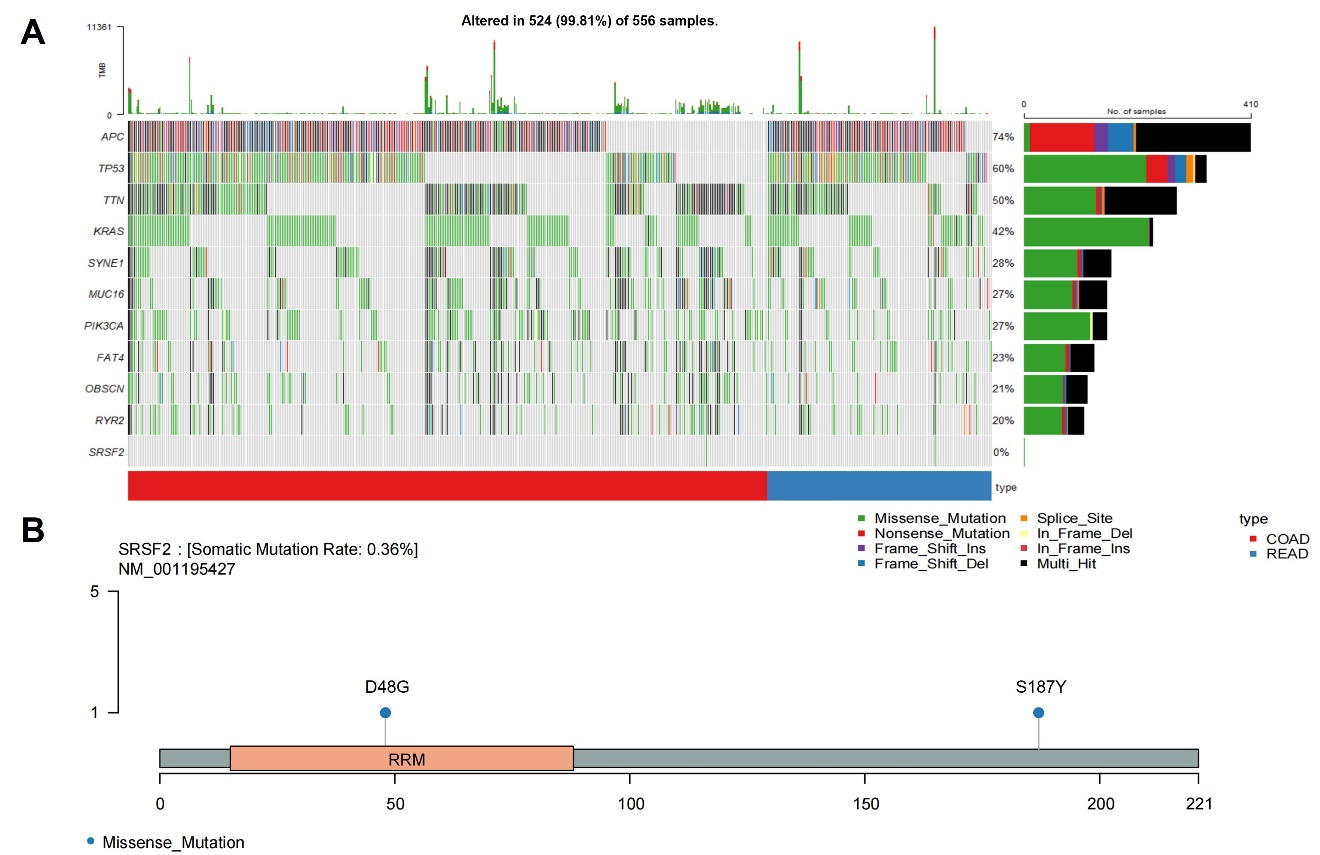
**

**Supplementary Figure 1. In silico analysis of SRSF2 mutation in CRC samples.** (A) Oncoplot displays the landscape of somatic mutations in CRC cohort from the TCGA datasets. Genes on the left are ordered by their mutation frequencies, and samples are ordered by disease histology, the annotation bar was on the bottom. The bar plot on the right shows the (-log10) transformed q-values, as estimated using MutSigCV algorithm. (B) Lollipop charts shows the somatic mutation rate and the mutation sites of SRSF2.

**Supplementary Figure 2**

**
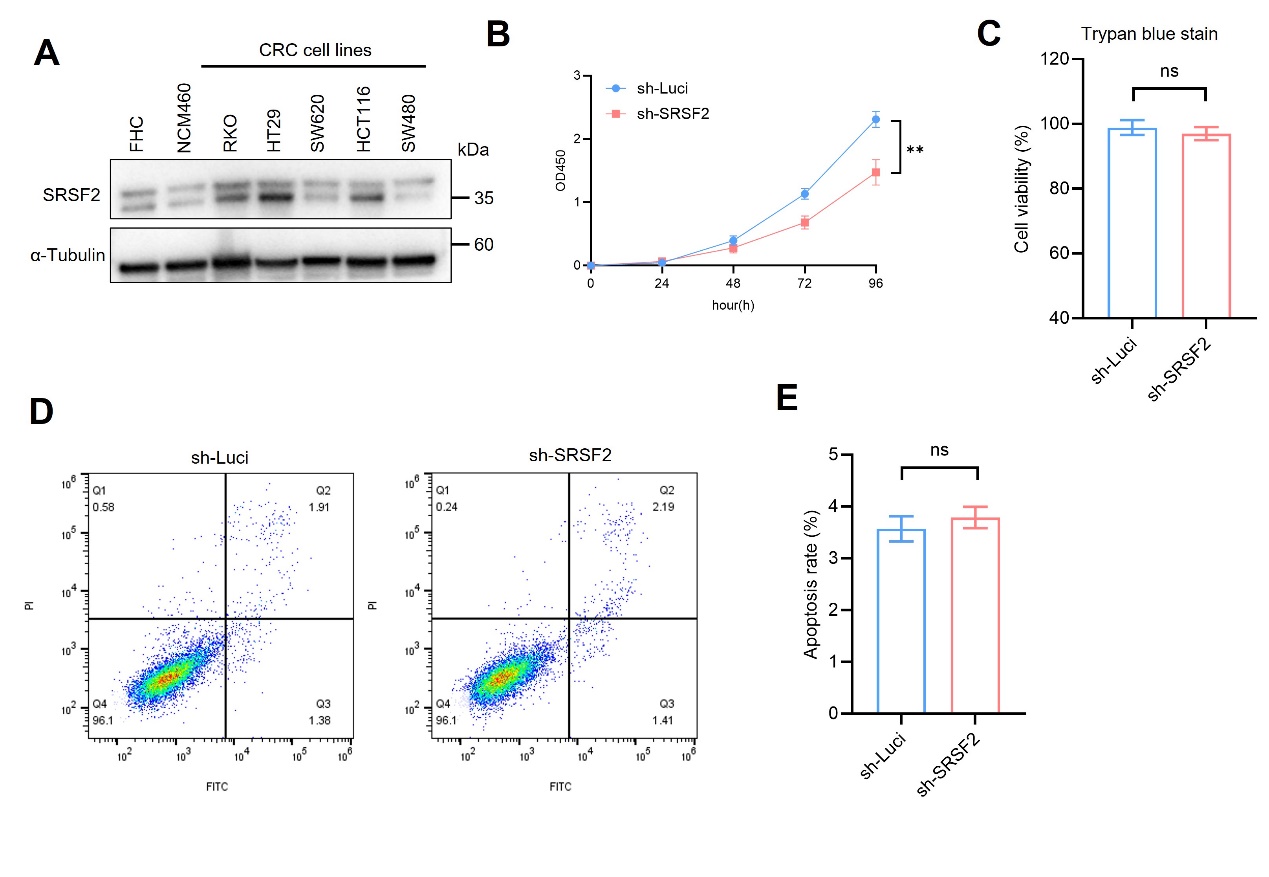
**

**Supplementary Figure 2. SRSF2 is highly expressed in CRC cell lines and has no significant effects on cell viability and apoptosis.** (A) Western blot of normal colon cells (NCM460 and FHC) and colon cancer cell lines (RKO, HT29, SW620, HCT116 and SW480) were performed using anti-α-tubulin and anti-SRSF2 antibodies, independently. (B) RKO cells were stably knockdown using lentiviruses transfected with SRSF2 shRNA (sh-SRSF2) or negative control shRNA (sh-Luci). CCK-8 proliferation assay was used to analyze cell proliferation of RKO cells with sh-SRSF2 and sh-Luci transfection. **P < 0.01. (C) Cell viability analysis were determined in RKO cells described in (B) by trypan blue staining. Data are presented as the mean ± S.D. ns: no significance. (D-E) Flow Cytometry Image (D) for cell apoptosis of RKO cells described in (B). Quantification of apoptosis was shown as mean ± SD in the bar graph (E). ns: no significance.

**Supplementary Figure 3**

**
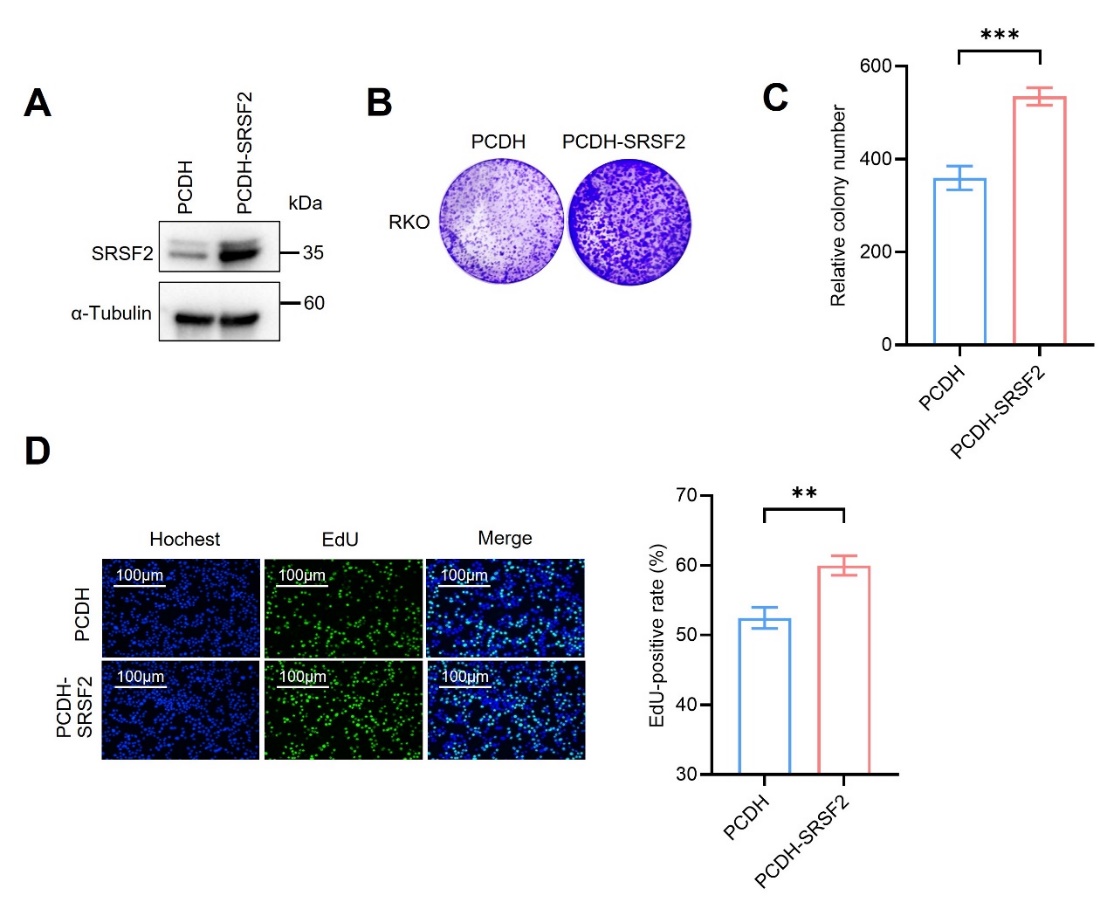
**

**Supplementary Figure 3. Overexpression of SRSF2 promotes proliferation of colon cancer cell.** (A) RKO cells were transfected with lentivirus-expressing PCDH-SRSF2 or PCDH empty vector control respectively, and selected for puromycin resistance. Western blot was performed using anti-α-tubulin and anti-SRSF2 antibodies independently. (B-C) Crystal violet staining with representative cells described in (A) were shown after clonogenic survival assay performed (B). The quantification of focal adhesions was shown as mean ± SD in the bar graph (C). *** P < 0.001. (D) The representative images of EdU staining assay for cells described in (A) were shown (Left). The results were presented as mean ± SD (Right). **P < 0.01.

**Supplementary Figure 4**

**
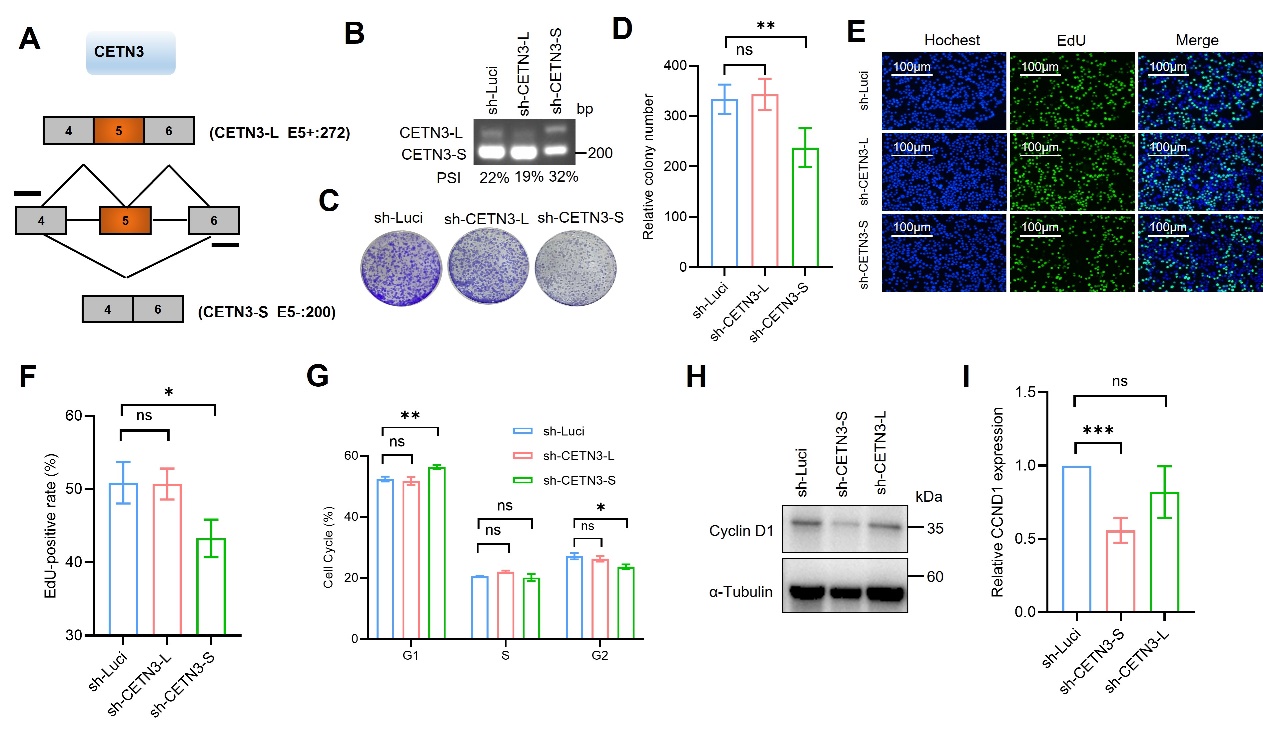
**

**Supplementary Figure 4. Knockdown of CETN3-S reduces the proliferation of colon cancer cell and induces G1 arrest in in cell cycle progression.** (A) Schematic diagram for detection of CETN3 splice variants containing or lacking alternative exon 5 (E5+ or E5-). The product sizes for two variants of CETN3 are shown. (B) RKO cells were stably knockdown using lentiviruses transfected with isoform-specific shRNAs, which targeted against the CETN3-L or CETN3-S variants independently. CETN3 knockdown efficiency using sh-CETN3-L or sh-CETN3-S compared with sh-Luci in colon cancer RKO cells was assessed by RT-PCR analysis. The quantification of PSI was shown under the RT-PCR results. (C-D) Crystal violet staining with representative cells described in (B) were shown after clonogenic survival assay performed (C). The quantification of focal adhesions was shown as mean ± SD in the bar graph (D). ns: no significance, ** P < 0.01. (E-F) The representative images of EdU staining assay for cells described in (B) were shown (E). The results were presented as mean ± SD (F). ns: no significance, *P < 0.05. (G) Cell cycle was analyzed in cells described in (B). The quantification of the representative DNA content was shown as mean ± SD in the bar graph. ns: no significance, *P < 0.05, **P < 0.01. (H) Western blot of cells described in (B) using anti-α-Tubulin and anti-Cyclin D1 antibodies independently. (I) Quantification of the western blot in (H). α-Tubulin is used to normalize the results, the relative CCND1 expression of control cells was set as 100%. The data represent three independent experiments, each value was shown as mean ± SD in the bar graph. ns: no significance, ***P < 0.001.

**Supplementary Figure 5**

**
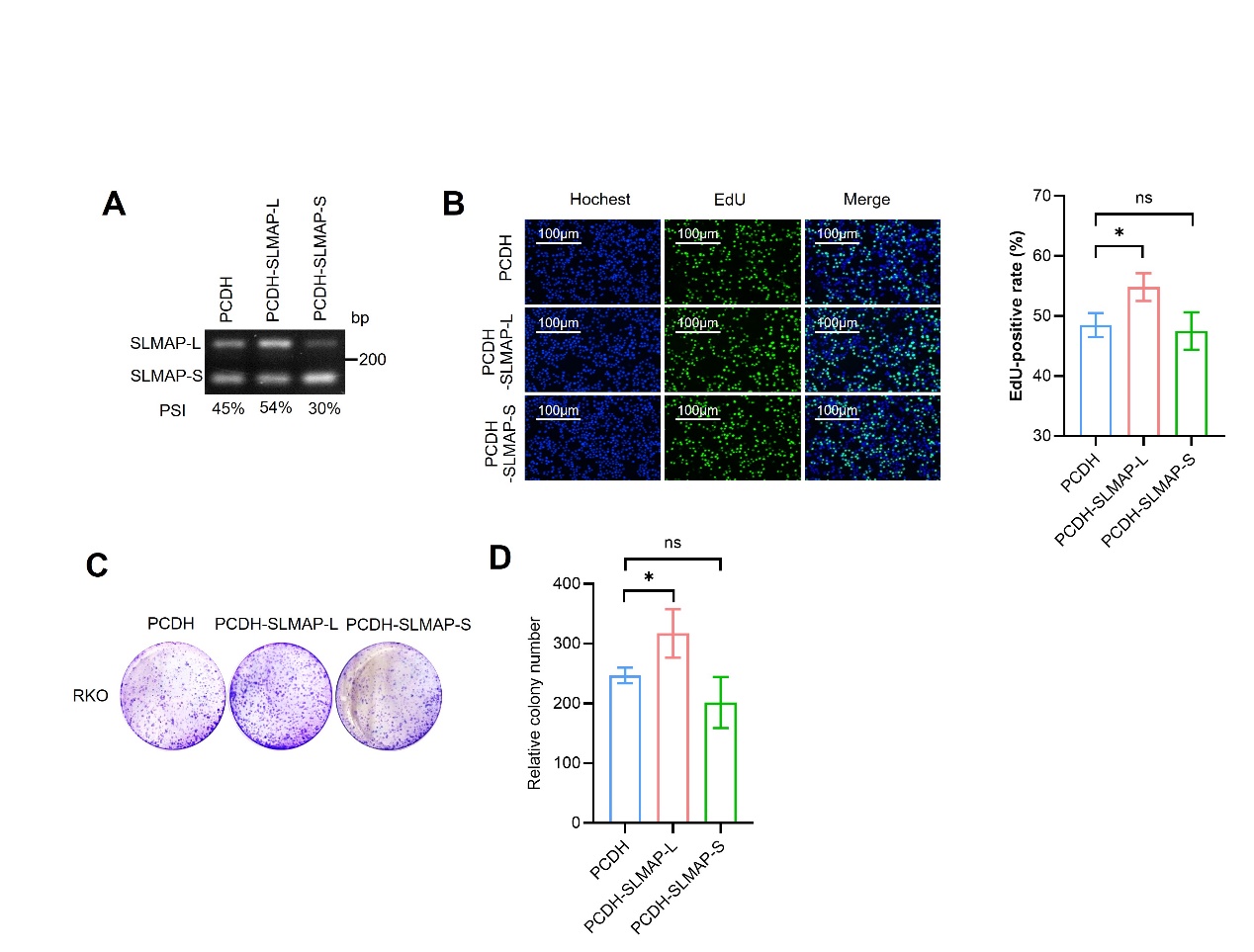
**

**Supplementary Figure 5. Overexpression of SLMAP-L splice isoform promotes proliferation of colon cancer cells.** (A) RKO cells were transfected with lentivirus-expressing PCDH-SLMAP-L, PCDH-SLMAP-S, or PCDH empty vector control, respectively, and selected for puromycin resistance. RT-PCR was performed to analyze the inclusion/skipping of SLMAP exon24. Each PSI value quantification from RT-PCR results was shown under the PCR image. (B) The representative images of EdU staining assay for cells described in (A) were shown (Left). The results were presented as mean ± SD (Right). ns: no significance, *P < 0.05. (C-D) Crystal violet staining with representative cells described in (A) were shown after clonogenic survival assay performed (C). The quantification of focal adhesions was shown as mean ± SD in the bar graph (D). ns: no significance, *P < 0.05.

**Supplementary Figure 6**

**
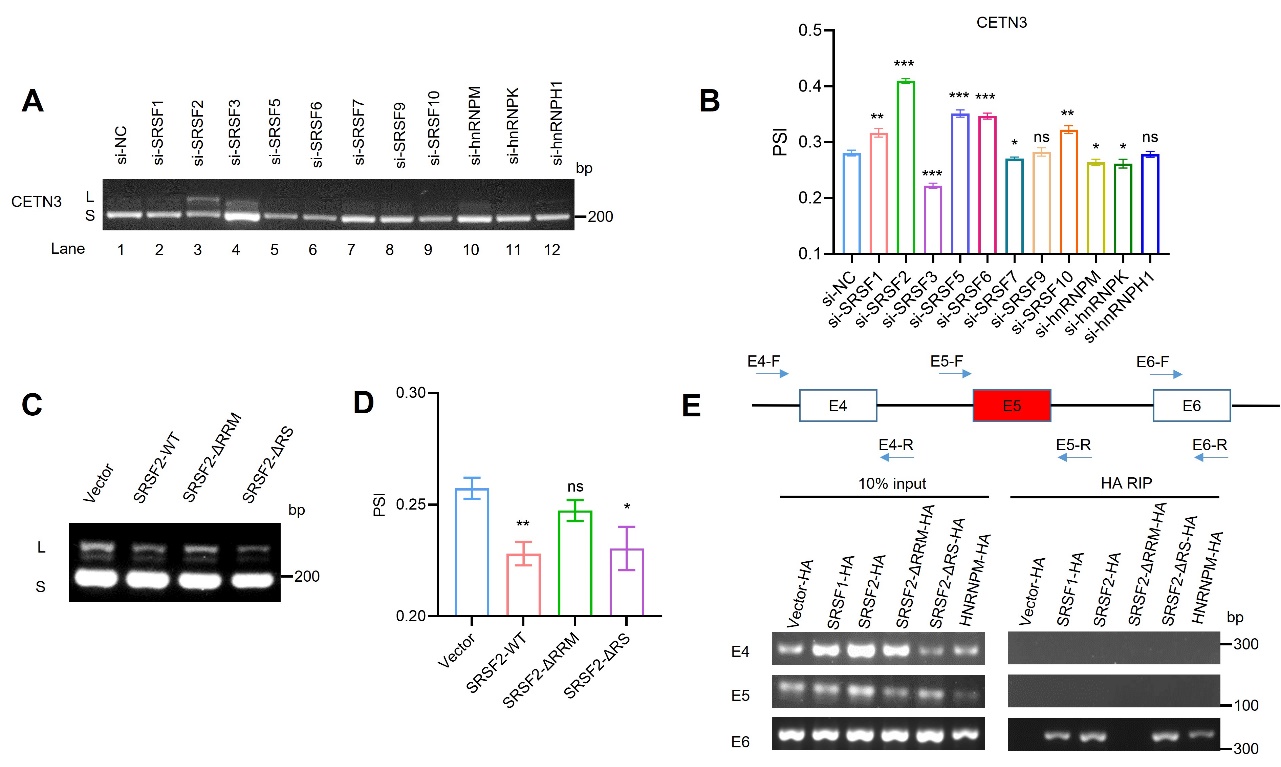
**

**Supplementary Figure 6. The binding between RRM domain in SRSF2 protein and endogenous CETN3 pre-mRNA fragments.** (A-B) The RT-PCR analysis of CETN3 exon 5 inclusion/skipping (A) after the knockdown of indicated SR or hnRNP proteins (A). The quantification of each PSI value from RT-PCR results of CETN3 splice variants was shown as mean ± SD in the bar graph (B). ns: no significance, *P < 0.05, **P < 0.01, ***P < 0.001. (C-D) The indicated plasmids were transiently transfected into RKO cells and RT-PCR was performed to analyze CETN3 exon5 inclusion/skipping (C). Each PSI value quantification from RT-PCR results was shown as mean ± SD in the bar graph (D). ns: no significance, *P < 0.05, **P < 0.01. (E) Upper: Diagram for the specific primers to detect exons 4-6 in CETN3 pre-mRNA. Bottom: CLIP assay, followed RT-PCR analysis indicated the direct binding between the indicated splicing regulators and endogenous CETN3 pre-mRNA fragments.

**Supplementary Figure 7**

**
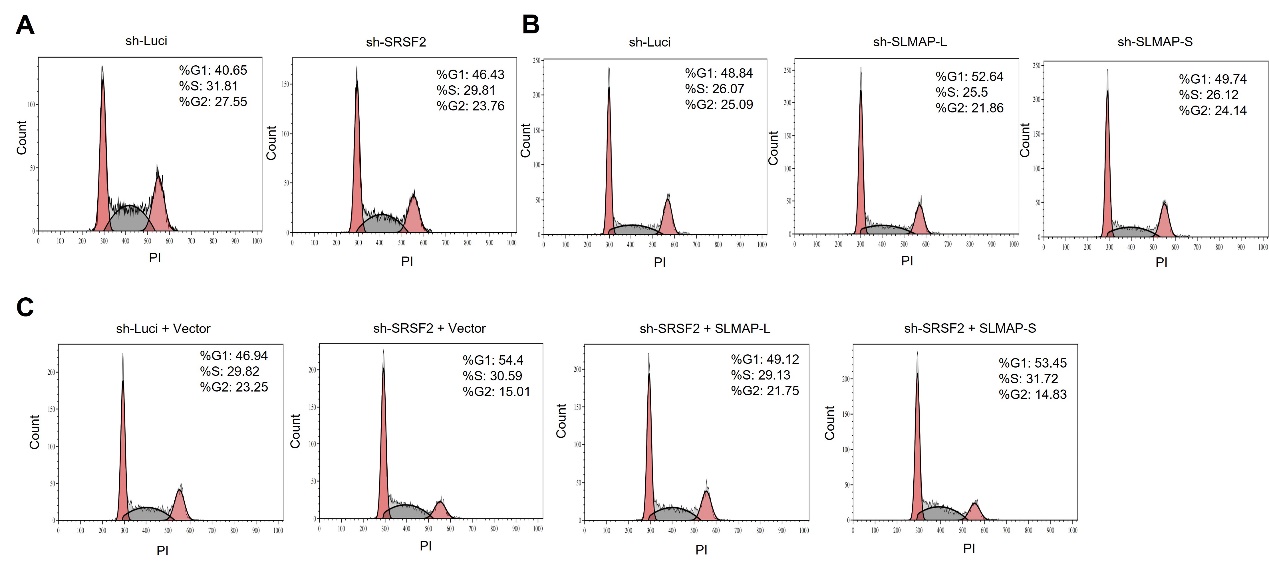
**

**Supplementary Figure 7. Knockdown of SRSF2 or SLMAP-L induces G1 arrest, and rescue of SLMAP-L in SRSF2 knockdown RKO cells partially reverses G1 arrest in cell cycle progression.** (A-B) Cell cycle was analyzed by flow cytometry in SRSF2 knockdown RKO cells (sh-SRSF2) and the control RKO cells (sh-Luci) shown in (A), SLMAP-L or SLMAP-S knockdown RKO cells (sh-SLMAP-L or sh-SLMAP-S) and the control RKO cells (sh-Luci) shown in (B). (C) Cell cycle was analyzed by flow cytometry in SRSF2 knockdown RKO cells stably transfected with SLMAP-L or SLMAP-S (sh-SRSF2 + SLMAP-L, sh-SRSF2 + SLMAP-S), SRSF2 knockdown RKO cells stably transfected with vector (sh-SRSF2 + Vector) and the control RKO cells (sh-Luci + Vector).

**Supplementary Figure 8**

**
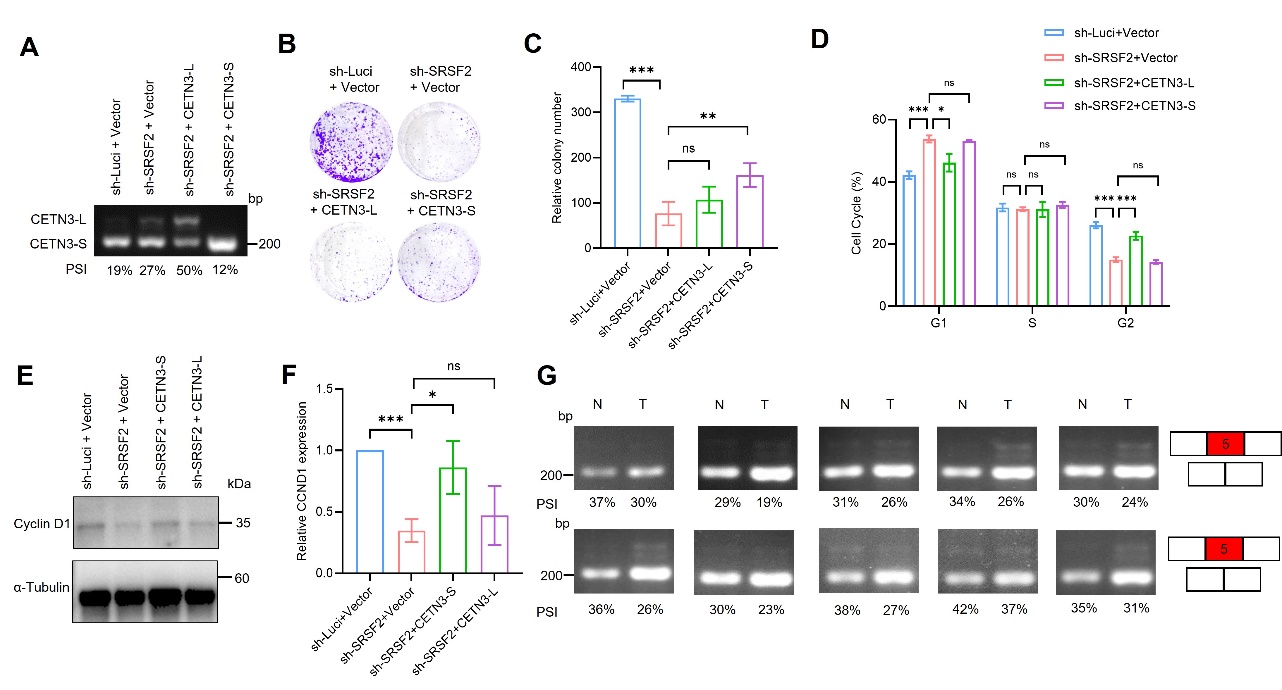
**

**Supplementary Figure 8. Abnormal splicing changes of CETN3 occur in CRC tumor samples and restoration of CETN3-S reverses SRSF2 knockdown-mediated phenotypes in colon cancer cells.** (A) RT-PCR was performed to analyze the inclusion/skipping of CETN3 exon5 in SRSF2 knockdown RKO cells stably transfected with CETN3-L or CETN3-S. Each PSI value quantification from RT-PCR results was shown under PCR results. (B-C) Clonogenic survival assay for cells described in (A) was performed and crystal violet staining with representative cells were shown (B). The relative number of focal adhesions described in (B) was quantified as mean ± SD in the bar graph (C). ns: no significance, **P < 0.01, ***P < 0.001. (D) Cell cycle was analyzed in cells described in (A). The quantification of representative DNA content was shown as mean ± SD in the bar graph. ns: no significance, *P < 0.05, ***P < 0.001. (E) Western blot of cells described in (A) using anti-α-tubulin and anti-Cyclin D1 antibodies independently. (F) Quantification of the western blot in (E). α-tubulin is used to normalize the results, the relative CCND1 expression of control cells was set as 100%. The data represent three independent experiments, each value was shown as mean ± SD in the bar graph. ns: no significance, *P < 0.05, ***P < 0.001. (G) Representative RT-PCR results for CETN3 splicing variants are shown between colorectal tumor samples (T) and matched normal tissues (N), and the PSI quantification of each splice variant was presented under the RT-PCR results. Note that alternative exon 5 in CETN3 was marked in red.

**Supplementary table 1. Clinical information of 51 CRC patients.**

| Patient Number | Gender | Age | Tumor site | T | N | M | TNM | Status |
| --- | --- | --- | --- | --- | --- | --- | --- | --- |
| 1 | Male | 78 | Rectum | 3 | 0 | 0 | IIA | Alive |
| 2 | Female | 55 | Colon | 3 | 2 | 1 | IIIC | Alive |
| 3 | Male | 62 | Rectum | 4a | 1 | 0 | IIIB | Alive |
| 4 | Male | 91 | Colon | 3 | 1 | 0 | IIIB | Alive |
| 5 | Male | 71 | Colon | 2 | 0 | 0 | I | Alive |
| 6 | Male | 53 | Rectum | 2 | 0 | 0 | I | Alive |
| 7 | Female | 75 | Colon | 3 | 2a | 0 | IIIB | Alive |
| 8 | Male | 71 | Rectum | 2 | 1c | 0 | IIIA | Alive |
| 9 | Female | 48 | Rectum | 1 | 0 | 0 | I | Alive |
| 10 | Female | 64 | Colon | 2 | 0 | 0 | I | Alive |
| 11 | Female | 63 | Rectum | 3 | 1 | 0 | IIIB | Alive |
| 12 | Female | 71 | Rectum | 3 | 0 | 0 | IIA | Alive |
| 13 | Female | 82 | Colon | 3 | 0 | 0 | IIA | Alive |
| 14 | Male | 68 | Colon | 4a | 0 | 0 | IIB | Alive |
| 15 | Male | 63 | Colon | 3 | 0 | 0 | IIA | Alive |
| 16 | Male | 72 | Rectum | 3 | 0 | 0 | IIA | Alive |
| 17 | Female | 64 | Colon | 2 | 0 | 0 | I | Alive |
| 18 | Male | 52 | Colon | 3 | 2b | 0 | IIIC | Alive |
| 19 | Male | 70 | Colon | 4 | 2b | 1c | IVC | Alive |
| 20 | Female | 71 | Colon | 3 | 1 | 0 | IIIB | Alive |
| 21 | Female | 70 | Rectum | 3 | 1 | 0 | IIIB | Alive |
| 22 | Male | 74 | Colon | 3 | 0 | 0 | IIA | Alive |
| 23 | Male | 58 | Rectum | 3 | 0 | 0 | IIA | Alive |
| 24 | Female | 49 | Colon | 3 | 1 | 0 | IIIB | Alive |
| 25 | Male | 80 | Rectum | 3 | 2b | 0 | IIIC | Alive |
| 26 | Male | 49 | Colon | 3 | 1b | 0 | IIIB | Alive |
| 27 | Male | 88 | Rectum | 3 | 2b | 0 | IIIC | Alive |
| 28 | Female | 66 | Rectum | 2 | 0 | 0 | I | Alive |
| 29 | Female | 85 | Rectum | 3 | 0 | 0 | IIA | Alive |
| 30 | Male | 55 | Rectum | 3 | 0 | 0 | IIA | Alive |
| 31 | Female | 72 | Rectum | 2 | 0 | 0 | I | Alive |
| 32 | Male | 58 | Colon | 4 | 2b | 0 | IIIC | Alive |
| 33 | Male | 85 | Rectum | 3 | 0 | 0 | IIA | Alive |
| 34 | Male | 48 | Rectum | 3 | 0 | 0 | IIA | Alive |
| 35 | Female | 38 | Rectum | 4 | 2 | 0 | IIIC | Alive |
| 36 | Female | 62 | Colon | 3 | 0 | 0 | IIA | Alive |
| 37 | Male | 54 | Colon | 4 | 0 | 0 | IIB | Alive |
| 38 | Male | 78 | Rectum | 1 | 1a | 0 | IIIA | Alive |
| 39 | Female | 59 | Colon | 3 | 0 | 0 | IIA | Alive |
| 40 | Male | 69 | Colon | 3 | 1 | 0 | IIIB | Alive |
| 41 | Male | 46 | Colon | 3 | 0 | 0 | IIA | Alive |
| 42 | Male | 74 | Rectum | 3 | 0 | 0 | IIA | Alive |
| 43 | Male | 64 | Rectum | 2 | 0 | 0 | I | Alive |
| 44 | Male | 68 | Rectum | 4 | 0 | 0 | II | Alive |
| 45 | Female | 68 | Rectum | 3 | 2b | 0 | IIIC | Alive |
| 46 | Male | 78 | Colon | 4 | 1c | 0 | IIIB | Alive |
| 47 | Female | 36 | Colon | 4 | 0 | 0 | II | Alive |
| 48 | Female | 66 | Colon | 4 | 1a | 0 | IIIB | Alive |
| 49 | Male | 66 | Colon | 3 | 0 | 0 | IIA | Alive |
| 50 | Female | 54 | Colon | 3 | 0 | 0 | IIA | Alive |
| 51 | Male | 58 | Rectum | 2 | 0 | 0 | I | Alive |

**Supplementary table 2. List of validated alternative splicing events affected by SRSF2**

| AccID | Description | delta_PSI | AS Type | Splicing Type | Junction reads (In::Ex) | | Location |
| --- | --- | --- | --- | --- | --- | --- | --- |
|  |  |  |  |  | KD2-RKO_Junc_Inclusive::Exclusive | NC-RKO_Junc_Inclusive::Exclusive |  |
| PAN3 | PAN3 poly(A) specific ribonuclease subunit | 0.430232558 | Exon out | Cassette | 25::25 | 6::80 | chr13:28239579-28239696 |
| CETN3 | centrin, EF-hand protein, 3 | 0.438460196 | Exon out | Cassette | 74::54 | 25::154 | chr5:90396493-90396564 |
| BCL2L1 | BCL2-like 1 | -0.096529887 | Exon in | Cassette | 1::521 | 38::348 | chr20:31666991-31667147 |
| GPAT4 | 1-acylglycerol-3-phosphate O-acyltransferase 6 | 0.157349896 | Exon out | Cassette | 33::128 | 12::240 | chr8:41619221-41619351 |
| PLEKHA3 | pleckstrin homology domain containing, family A (phosphoinositide binding specific) member 3 | 0.376618572 | Exon out | Cassette | 21::32 | 1::50 | chr2:178488968-178489014 |
| RPE | ribulose-5-phosphate-3-epimerase | 0.299159664 | Exon out | Cassette | 46::94 | 7::231 | chr2:210018153-210018221 |
| XPA | xeroderma pigmentosum, complementation group A | -0.437973485 | Exon in | Cassette_multi | 3::61 | 32::34 | chr9:97682200-97682430 |
| SNF8 | SNF8, ESCRT-II complex subunit | -0.07463162 | Exon in | Cassette | 1::246 | 31::363 | chr17:48936855-48936933 |
| NAV2 | neuron navigator 2 | 0.327782056 | Exon out | Cassette | 104::92 | 43::169 | chr11:20056530-20056628 |
| DDHD1 | DDHD domain containing 1 | 0.348280296 | Exon out | Cassette | 79::23 | 26::35 | chr14:53051844-53051927 |
| EIF4H | eukaryotic translation initiation factor 4H | 0.202380952 | Exon out | Cassette | 322::350 | 310::810 | chr7:74190247-74190306 |
| GPBP1 | GC-rich promoter binding protein 1 | 0.395087719 | Exon out | Cassette | 190::60 | 104::181 | chr5:57237113-57237172 |
| CARM1 | coactivator-associated arginine methyltransferase 1 | 0.307246616 | Exon out | Cassette | 134::65 | 67::116 | chr19:10921375-10921443 |
| PARD3 | par-3 family cell polarity regulator | -0.321535262 | Exon in | Cassette | 34::181 | 177::192 | chr10:34372498-34372536 |
| FXR1 | fragile X mental retardation, autosomal homolog 1 | -0.19818937 | Exon in | IR | 64::223 | 174::239 | chr3:180962940-180963028 |
| CSNK1A1 | casein kinase 1, alpha 1 | 0.111190273 | Exon out | Cassette | 143::495 | 82::644 | chr5:149517794-149517877 |
| MRPL1 | mitochondrial ribosomal protein L1 | -0.31027001 | Exon in | Cassette | 7::115 | 25::43 | chr4:77906946-77907210 |
| SPTAN1 | spectrin, alpha, non-erythrocytic 1 | -0.110169492 | Exon in | Cassette | 0::242 | 39::315 | chr9:128609651-128609665 |
| PARP4 | poly (ADP-ribose) polymerase family, member 4 | 0.194538887 | Exon out | Cassette | 30::74 | 17::164 | chr13:24432581-24432696 |
| SLMAP | sarcolemma associated protein | -0.392758143 | Exon in | Cassette | 16::58 | 95::61 | chr3:57925845-57925934 |
| VAPA | VAMP (vesicle-associated membrane protein)-associated protein A, 33kDa | 0.106660153 | Exon out | Cassette | 81::364 | 52::638 | chr18:9944924-9945058 |
| VPS39 | vacuolar protein sorting 39 homolog (S. cerevisiae) | 0.328095975 | Exon out | Cassette | 62::23 | 61::91 | chr15:42192066-42192098 |
| HSF1 | heat shock transcription factor 1 | 0.366274219 | Exon out | Cassette | 171::30 | 78::83 | chr8:144312628-144312711 |
| SNX14 | sorting nexin 14 | -0.149279098 | Exon in | Cassette | 13::171 | 64::227 | chr6:85538838-85538864 |
| MAP3K4 | mitogen-activated protein kinase kinase kinase 4 | -0.36205074 | Exon in | Cassette | 3::40 | 19::25 | chr6:161098278-161098427 |
| ADGRB2 | brain-specific angiogenesis inhibitor 2 | 0.362068966 | Exon out | Cassette | 25::25 | 12::75 | chr1:31735183-31735281 |
| CDK11B | cyclin-dependent kinase 11B | 0.24 | Exon out | A3SS | 6::19 | 0::82 | chr1:1645262-1645289 |
| IFI16 | interferon, gamma-inducible protein 16 | -0.098214286 | Exon in | Cassette | 101::11 | 285::0 | chr1:159049432-159049599 |
| TXNL4A | thioredoxin-like 4A | 0.133226324 | Exon out | Cassette | 110::335 | 71::552 | chr18:79976718-79976787 |
| OPA1 | optic atrophy 1 (autosomal dominant) | 0.237829552 | Exon out | Cassette | 112::121 | 68::212 | chr3:193617784-193617837 |
| COL4A3BP | collagen, type IV, alpha 3 (Goodpasture antigen) binding protein | 0.124208145 | Exon out | Cassette | 31::125 | 19::236 | chr5:75399310-75399387 |
| LSR | lipolysis stimulated lipoprotein receptor | -0.116504854 | Exon in | Cassette | 0::100 | 12::91 | chr19:35261925-35261981 |
| COPE | coatomer protein complex, subunit epsilon | -0.093055186 | Exon in | Cassette | 2::157 | 30::254 | chr19:18905867-18905935 |

**Supplementary table 3. The siRNAs used in this study.**

| Names | Sequences (5’-3’) |
| --- | --- |
| siNC | UUCUUCGAACGUGUCACGUTT |
| si-SRSF1 | AGGACAUUGAGGACGUGUUTT |
| si-SRSF2 | UCCAAAUCCAGGUCGCGAUTT |
| si-SRSF3 | CCUGUCCAUUGGACUGUAATT |
| si-SRSF5 | GGUUGAGUUUGCCUCUUAUTT |
| si-SRSF6 | CCUCGAAGUAGACCUCAAATT |
| si-SRSF7 | CCUCGACGAUCAAGAUCUATT |
| si-SRSF9 | GAGGACCUGUUCUACAAGUTT |
| si-SRSF10 | GCGUGAAUUUGGUCGUUAUTT |
| si-hnRNPM | GCCAGAUAUUUGUGAGAAATT |
| si-hnRNPK | UGGAAACUGAACAGCCAGATT |
| si-hnRNPH1 | GGGACACAGAUAUGUUGAATT |

**Supplementary table 4. The shRNAs used in this study.**

| Names | Sequences (5’-3’) |
| --- | --- |
| sh-Luci-F | CCGGTTCCTGGAACAATTGCTTTTACTCGAGTAAAAGCAATTGTTCCAGGAATTTTTG |
| sh-Luci-R | AATTCAAAAATTCCTGGAACAATTGCTTTTACTCGAGTAAAAGCAATTGTTCCAGGAA |
| sh-SRSF2#1-F | CCGGAACCGCACTCGTTCTCGATCTCTCGAGAGATCGAGAACGAGTGCGGTTTTTTTG |
| sh-SRSF2#1-R | AATTCAAAAAAACCGCACTCGTTCTCGATCTCTCGAGAGATCGAGAACGAGTGCGGTT |
| sh-SRSF2#2-F | CCGGAACCAGTGTCCAAGAGGGAATCTCGAGATTCCCTCTTGGACACTGGTTTTTTTG |
| sh-SRSF2#2-R | AATTCAAAAAAACCAGTGTCCAAGAGGGAATCTCGAGATTCCCTCTTGGACACTGGTT |
| sh-SLMAP-L-F | CCGGAACATATTACAACCCGTCCCACTCGAGTGGGACGGGTTGTAATATGTTTTTTTG |
| sh-SLMAP-L-R | AATTCAAAAAAACATATTACAACCCGTCCCACTCGAGTGGGACGGGTTGTAATATGTT |
| sh-SLMAP-S-F | CCGGAAAGGAAATAATAAACCCTGGCTCGAGCCAGGGTTTATTATTTCCTTTTTTTTG |
| sh-SLMAP-S-R | AATTCAAAAAAAAGGAAATAATAAACCCTGGCTCGAGCCAGGGTTTATTATTTCCTTT |
| sh-CETN3-S-F | CCGGAAGTGATGGAGAAATAAACCACTCGAGTGGTTTATTTCTCCATCACTTTTTTTG |
| sh-CETN3-S-R | AATTCAAAAAAAGTGATGGAGAAATAAACCACTCGAGTGGTTTATTTCTCCATCACTT |
| sh-CETN3-L-F | CCGGAAGTGATGGAGAAATTCTTAACTCGAGTTAAGAATTTCTCCATCACTTTTTTTG |
| Sh-CETN3-L-R | AATTCAAAAAAAGTGATGGAGAAATTCTTAACTCGAGTTAAGAATTTCTCCATCACTT |

**Supplementary table 5. The primers used in this study.**

| Names | Sequences |
| --- | --- |
| RPE-F | TGAGGACCCAGTTCCCATCT |
| RPE-R | AGGGAGATTTCATGAACAGGAACA |
| HSF1-F | CCCTCATTGACTCCATCCTG |
| HSF1-R | TCAGCATGGTCTGCAGGTTA |
| GPBP1-F | TGGATGGCGTACACATGGAA |
| GPBP1-R | TTTGTAGGTGGAGCAGCAGG |
| FXR1-F | CTGGTGTGGTTCGAGTGAGA |
| FXR1-R | CCGCCTACGACGGTTAGTAC |
| SLMAP-F | GCTGCTCCGAGAGAAAGGAA |
| SLMAP-R | AGCTTCTAGAGGGAGGACGG |
| SNX14-F | TTGCTGAAGGCCCATACATA |
| SNX14-R | CCAAATGATTCTCCCCTTTTC |
| OPA1-F | GATCAGTGGAAAGATATGATACCG |
| OPA1-R | ATGCTTGTCACTTTCAGATCCA |
| MAP3K4-F | ACCTCGATGCCATAGTGACC |
| MAP3K4-R | TGGACCCACTTGAATCTCCT |
| PAN3-F | CCTCCAGGCTGAGTAACGTG |
| PAN3-R | AGGTGCTGGAGTTGTGTCTG |
| PLEKHA3-F | GCAGCCTCGTTGGTTTGTTT |
| PLEKHA3-R | GCCACCTCTGTCTTTCAGCT |
| NAV2-F | AGATGAGCTTGTCCAACCCG |
| NAV2-R | TGACGATGTGCTGGAAACCA |
| EIF4H-F | ATCGGTCACTTCGTGTGGAC |
| EIF4H-R | CATGTTGGATCCACGGAGGG |
| CETN3-F | TGGAAAGAGATCCCCATGAAGA |
| CETN3-R | AGCAATGAACTCCTCTTGGTT |
| β-actin-F | GCAAAGACCTGTACGCCA |
| β-actin-R | TGCATCCTGTCGGCAATG |
| SRSF1-F | GCGACGGCTATGATTACGAT |
| SRSF1-R | CTTGGAGGCAGTCCAGAGAC |
| SRSF2-F | CTACAGCCGCTCGAAGTCTC |
| SRSF2-R | TTGGATTCCCTCTTGGACAC |
| SRSF3-F | AACAAGACGGAATTGGAACG |
| SRSF3-R | TGGGCCACGATTTCTACTTC |
| SRSF5-F | CAAGAAGCAGGTCTCGATCC |
| SRSF5-R | CCACGTTTCTGGCTCTTCTC |
| SRSF6-F | AAGCGTGCTTTGGACAAACT |
| SRSF6-R | TCCTGCGACTCCTACTTCGT |
| SRSF7-F | AGCATCTCCTCGACGATCA |
| SRSF7-R | CTGCTTCTTGGTCGTGAAAT |
| SRSF9-F | AGGAATGGGCCTCCTACAAG |
| SRSF9-R | GCAGGGCATATTCCATGTCT |
| SRSF10-F | GAGGATGTTCGTGATGCTGA |
| SRSF10-R | CCGACTTCTTGATCTCCTCCT |
| hnRNPK-F | GCCCTGCAGAAGATATGGAA |
| hnRNPK-R | CACTGCTGTCTGGGACTGAA |
| hnRNPM-F | AGGAAGGCCTGCCAGATATT |
| hnRNPM-R | CTGGCGACTCGAACTTAACC |
| hnRNPH1-F | TCCAGAGCACAACAGGACAC |
| hnRNPH1-R | CGAACTCGACATCTGCTTCA |
| SLMAP-E23-F | ATTTTAAGTTGTATCTGCACACTTTTT |
| SLMAP-E23-R | AGCCAAGTTTGCAAAGACTTAC |
| SLMAP-E24-F | TGATTTTAATATGCCTTCCCTTCTTT |
| SLMAP-E24-R | CGGGGACAAACAGACAAGGA |
| SLMAP-E25-F | GGTTGCAGTAACAGCCATCG |
| SLMAP-E25-R | AGCTTCTAGAGGGAGGACGG |
| SLMAP-I23-F | AGGACTGCCTGGAGGAAGAG |
| SLMAP-I23-R | AGCAGGGGAGAAACAAGAGC |
| SLMAP-I24-F | CCAGGACTTTCTTGGGACGT |
| SLMAP-I24-R | ACCCCTTGGTTCCAGAGAGT |
| CETN3-E4-F | TGCCTTTGTGATTATGCAGGT |
| CETN3-E4-R | TGTAGCATCAGGAAAACCTGGA |
| CETN3-E5-F | TGTAATCAAATCATTCTAAGGCTGTT |
| CETN3-E5-R | TCAGTTTAGCCAGTGTGCAC |
| CETN3-E6-F | AACCAAGAGGAGTTCATTGCT |
| CETN3-E6-R | AGTCTGGAAAATGTGTGCACT |

**Supplement table 6. The antibodies used in this study.**

| Names | Manufacturers | Cat. Number |
| --- | --- | --- |
| Anti-SC35 antibody [EPR12238] | Abcam | ab204916 |
| SF2/ASF antibody (96) | Santa Cruz | SC-33652 |
| Anti-hnRNP M1-M4 antibody [EPR13509(B)] | Abcam | ab177957 |
| Anti-HA tag antibody - ChIP Grade | Abcam | ab9110 |
| Alpha Tubulin Polyclonal antibody | Proteintech | 11224-1-AP |
| CCND1 Antibody | BOSTER | BST17044272 |
| CDK6 Antibody | BOSTER | BOS778BP98 |
